# The Ukraine war and rising commodity prices: Implications for developing countries

**DOI:** 10.1016/j.gfs.2023.100680

**Published:** 2023-03

**Authors:** Channing Arndt, Xinshen Diao, Paul Dorosh, Karl Pauw, James Thurlow

**Affiliations:** International Food Policy Research Institute, Washington D.C, USA

**Keywords:** Global crisis, Ukraine, Poverty, Food security, Economywide models

## Abstract

The Russia-Ukraine war's impact on food, fuel, and fertilizer prices is a major concern for global poverty and food insecurity. Despite numerous studies and editorials on the risks and challenges of the crisis, there is little quantitative analysis of its consequences for developing countries. We use national economywide models to measure the near-term impacts of the crisis on agrifood systems, poverty, and food insecurity in 19 countries. Despite wide variations across countries, results confirm the adverse impacts of the crisis, with a total 27.2 and 22.3 million more people pushed into poverty and hunger, respectively. Agrifood systems and poverty are more vulnerable to rising fuel and fertilizer prices, whereas hunger and diet quality are more affected by higher food prices.

## Highlights

• The Ukraine War severely disrupted global markets for food, fuel, and fertilizer.
• The macroeconomic effects of rising prices depend on countries' unique economic structures.
• Socioeconomic impacts reflect countries' and individuals' consumption and employment patterns.
• Household consumption and poverty are most affected by higher fuel and food prices.
• Deteriorating undernourishment and diet quality are driven more by rising food prices.

## 1 Introduction

Global food, fuel, and fertilizer prices rose rapidly in the first half 2022, driven in large part by the fallout from the war in Ukraine and sanctions imposed on Russia. Other factors, such as fertilizer export bans, also contributed to global market disruptions (Hebebrand and Laborde 2022), alongside ongoing supply chain disruptions resulting from Covid-19. The ensuing global crisis raised concerns about the impacts of higher world commodity prices on developing countries, especially on global poverty and food insecurity (see, for example, Bentley et al., 2022; Economist 2022; Glauber and Laborde 2022). Most studies linking the crisis to food security focus on food prices (see, for example, WFP 2022). This overlooks the compounding impacts of rising fuel and fertilizer prices on food systems, as well as the economywide spillover effects that cause food insecurity to spread throughout populations. To effectively respond to the global crisis, governments and the development community need information on vulnerable countries and populations. In this paper we use national economywide models to assess the poverty and food security impacts of rising world prices in 19 developing countries.^1^

**Fig. 1:**
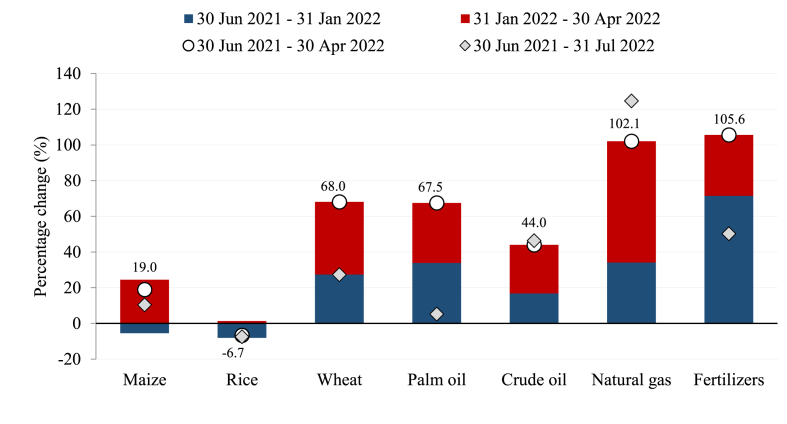
Changes in world commodity prices since mid-2021. Note: Figure reports changes in nominal prices in US dollars. Our modeling analysis converts these to real price changes, by deflating using the US Consumer Price Index, to account for an overall increase in world prices.

Source: Authors' calculations using World Bank commodity price data (World Bank 2022).

Unlike the 2008/09 food crisis, the price increases in 2022 were concentrated amongst a few specific commodities, mostly those with supply chains linked to Ukraine. This makes it more difficult to generalize about impacts across countries, given that countries have unique production and trade patterns. Fig. 1 shows price changes for key food and nonfood commodities between June 2021 and July 2022 (World Bank 2022). By April 2022, the world crude price had increased by almost a half; palm oil and wheat prices had risen by two-thirds; and natural gas and fertilizer prices had more than doubled. In contrast, the price of maize and rice – key staple crops in Africa and Asia – were less affected by the crisis, with world rice prices falling over the year.

Most increases in world prices occurred after the start of the Ukraine war in early February 2022 (see red bars in the figure). However, fertilizer prices had risen substantially prior to the start of the war, largely due to China imposing an export ban (Hebebrand and Laborde 2022). Given difficulties in attributing price changes to specific events and our focus on the price shocks facing developing countries in the first semester of 2022, we evaluate the impacts of world price changes from mid-2021 to April 2022. Analyses by the authors were distributed to decision-makers in developing countries and interested institutions globally in Q2 2022 using these shock vectors. We elect to continue to use these shock vectors for this article as they represent the information set coming available to policymakers at a key decision point in time. In the event, Fig. 1 shows that world prices for many commodities declined after in April 2022 (see diamond markers in the figure). By the end of July, however, wheat and fertilizer prices were still well above their June 2021 levels, and crude oil and natural gas prices remained close to peak levels.

Before proceeding, it merits mentioning that these higher global prices are a signal of penury. Because of the Ukraine war, supplies of foods, fuels, and fertilizers on global markets were appreciably lower in late April 2022 than they would otherwise have been. Further, at that point in time, the trajectory of the crisis was far from clear, further aggravation was a distinct possibility. Overall, this reduction in supply implies that the world was less well off with high prices for affected commodities signaling a need to reduce use/consumption as well as stimulate supply/availability where possible. In many locations, the terms of trade shifts also constituted a significant negative macroeconomic shock with substantial implications for balance of payments and knock-on implications for production, trade, employment, and ultimately welfare (in many dimensions).

This paper seeks to sort through these multi-faceted implications for 19 developing countries. It is structured as follows. Section 2 describes the models and data. Section 3 reports our estimated impacts on economies and food systems while section 4 focuses on poverty and food insecurity. The final section summarizes our finding, discusses implications, and proposes future research. .

## 2 Measuring impacts of the global crisis

### 2.1 Economywide models and data

Economywide models are used to assess the impacts of global price shocks on production, workers, and populations in 19 developing countries. More specifically, we use the International Food Policy Research Institute's Rural Investment and Policy Analysis (RIAPA) modeling system. At the core of RIAPA are Computable General Equilibrium (CGE) models, which capture all producers (sectors) and consumers (households) in an economy and track how they interact with each other in markets for products and factors (i.e., land, labor, and capital) to determine prices, incomes, and expenditures (see Diao and Thurlow 2012). RIAPA's main database is called a Social Accounting Matrix (SAM), which combines and reconciles data from a range of farm, firm, and household surveys, as well as data on domestic production, international trade, and government revenues and expenditures.

RIAPA captures many of the country-specific factors driving the impacts of the global crisis. Each economy is separated into detailed subsectors. Half of these are in agriculture, agro-processing, and food services, allowing us to track food supply chains and price shocks from farmers to consumers, and vice-versa. The models also separate fertilizers and fuels and track their use by consumers and as production inputs (e.g., fertilizers for farming or fuels for transport). Information on the flow of goods and services between sectors and to final users is drawn from supply-and-use tables embodied within the SAMs. Producers in the models maximize profits by (i) raising prices, subject to demand constraints; (ii) favoring cheaper inputs, including labor, land, and capital; and (iii) deciding whether to supply domestic or export markets.

RIAPA also separates countries into population groups using national household surveys. Workers are divided across rural and urban areas and by education levels, and households are grouped based on their rural-urban location, farm-nonfarm status, and per capita consumption quintile. This allows RIAPA to track which workers are most affected by the global crisis and which households are most affected by changes in wages, employment, and revenues from farm and nonfarm enterprises. Households in the models maximize welfare (consumption) by demanding relatively cheaper products, including switching between imports and domestic products.

The model and country data determine price transmission from global to local markets. The share of imports (exports) in total supply (demand) is a key factor. For example, countries that import fuels will experience larger domestic price increases when world prices rise than will countries that produce their own fuels. However, countries that export fuels may also experience rising domestic prices, as more domestic output is redirected to export markets. Price transmission also depends on the ability of producers and consumers to switch between domestic and foreign products/markets. This is represented in the models by product-specific trade substitution elasticities, which are drawn from the Global Trade Analysis Project (Dimaranan 2006).^2^ Elasticities are usually higher when domestic and foreign are more homogenous, such as wheat and petroleum. Consumer responses to changes in real incomes are also based on elasticities, which are estimated using national household surveys or drawn from the literature.

Finally, we make assumptions in the models to better reflect the short-run constraints that countries face in responding to global price shocks. First, we assume that central banks are not positioned to accommodate the price shocks through changes in foreign currency reserves. Second, farmers cannot reallocate crop land, but, instead, receive windfall revenues from higher prices based on pre-crisis land allocations. Similarly, producers of fertilizers and fuels cannot increase output, but do receive windfall gains. Third, we allow underemployment amongst less-educated workers to increase when economies contract, which is consistent with labor-markets being demand-driven in the short-run. Finally, we focus on the impacts of the global shocks without accounting for actual or potential policy responses to mitigate adverse impacts. These assumptions capture the rapid onset of the global crisis, reflect structural constraints, and are consistent with our focus on measuring short-run impacts at the peak of the global price increases.

In summary, our models are calibrated to detailed databases, built using official national accounts and nationally representative household surveys. The models provide a consistent representation of each country's unique production, trade, and consumption patterns, as well as the generation and distribution of incomes. The models use behavioral functions, grounded in economic theory, to represent producer and consumer behavior while respecting all macroeconomic identities. These functions are calibrated using elasticities estimated from household surveys or global databases. These models have been used previously to estimate the welfare implications of rising world food and fuel prices (e.g., Arndt et al., 2008); to evaluate the impacts of farmers' fertilizer use (Arndt et al., 2016); and to decompose historical drivers of poverty (Arndt et al., 2012). The models are therefore well-suited to evaluating the impacts of the recent food, fuel, and fertilizer crisis.

### 2.2 Baseline food and fuel supply patterns

The impact of rising world commodity prices in the models depends on countries' unique production, trade, and consumption patterns. Although maize, wheat, and edible oils are consumed in all the countries included in our analysis, the reliance on imports varies by crop and by country. African countries are broadly self-sufficient in maize, the continent's major staple food (Van Ittersum et al., 2016). However, most of the 14 African countries included in our analysis rely on imported wheat grains since this crop is not widely grown. Households in Africa also consume relatively few foods derived from wheat. Exceptions include Egypt, where bread is the most important staple, and Ethiopia, where wheat makes up a larger share than maize in the food basket and is produced at scale domestically.

World rice prices declined slightly between mid-2021 and April 2022, which will have benefited consumers in the five Asian countries included in our analysis, since rice is the major staple food (Adjao and Staatz 2015). Most of these Asian countries are also large producers of rice and are not overly reliant on imports. Rice is also more widely consumed than wheat in many African countries, including the Democratic Republic of the Congo (DRC), Ghana, and Senegal. Although rice is an important crop in Ghana and Senegal, most domestic supply still comes from imports. Falling world prices are therefore more likely to benefit consumers in these two countries.

In most countries, the share of edible oil imports – mostly palm oil – in domestic supply is smaller than wheat imports but larger than maize imports. On the other hand, imported edible oil products are close substitutes for domestically produced and consumed edible oils. Many countries grow various oilseeds and some export oilseeds, including Ethiopia, Tanzania, and Uganda. Therefore, the net effect of global food price movements is not immediately evident for many countries.

Turning to fuels, almost all oil products (i.e., crude oil and processed petroleum) are imported in the studied countries. Exceptions include Nigeria, which is one of Africa's largest oil exporters, as well as Egypt and Ghana, both of which export natural gas, crude oil, or refined petroleum. Other countries, such as DRC, Niger, and Uganda, produce crude oil at a small scale, but still mainly rely on imports. While crude oil producers in exporting countries will benefit from rising global oil prices, the impact of higher oil and petroleum prices on other sectors of their economies is ambiguous. Moreover, the impact of higher oil prices on households is harder to assess as direct consumption of oil products is limited. Instead, oil products are primarily used as an intermediate input into the production of other goods and services. For example, input use accounts for around 70–90 percent of total demand for oil products, with significant demand from the transport sector. Fuel price increases, therefore, indirectly affect the prices of all marketed goods and services in the economy.

The RIAPA model tracks the flow of domestic and imported inputs between sectors and estimates the net effect on final product prices. Impacts on households also depend on the importance of the affected commodities in their consumption baskets. Shares of cereals and edible oils in total food expenditure vary significantly across countries (i.e., 15–35 percent). Root crops are another important staple food in most countries, allowing consumers to switch to these nontraded foods when cereals prices rise. The RIAPA models measure incomes and food and nonfood expenditures for different population groups, and link to survey-based microsimulation models to track changes in consumption patterns of individual households. Unpacking populations is crucial since household spending patterns vary across population subgroups. Cereals and edible oils, for example, are often important in the consumption baskets of poorer or rural households, and so these households are expected to be adversely affected by higher prices for these products.

### 2.3 Baseline fertilizer use and farm productivity

Unless existing fertilizer subsidy programs are designed to cushion the effect of price shocks, rising fertilizer prices will likely cause some farmers to reduce their use of this input, leading to lower agricultural production and higher food prices. The magnitude of this decline depends on: (i) the amount of fertilizer currently used to grow crops (i.e., fertilizer adoption and application rates); (ii) the responsiveness of fertilizer demand to changes in prices (i.e., the price elasticity of demand for fertilizer); and (iii) the expected productivity losses for farmers who lower their fertilizer application rates. Our analysis captures both the more immediate impact of higher fertilizer prices on farm production costs, and the medium-term effects of higher prices on fertilizer use, and hence farm productivity.

Information on fertilizer use by crop was calculated using national farm or household survey data for most countries, while, for the few countries that lacked such data, we relied on global data (Ludemann et al., 2022) and local expert assessments. For Kenya and Nigeria, we used the International Fertilizer Development Center's (IFDC) Fertilizer Use by Crop (FUBC) estimates (see Africa Fertilizer, 2022). Together, these data confirm that fertilizer adoption rates vary widely across countries.^3^ Farmers in Asian countries, such as Bangladesh and Cambodia, tend to have higher adoption rates than their African counterparts. Adoption rates also vary among African countries, with relatively high adoption in Ethiopia and very low adoption in Uganda. Finally, adoption rates vary across crops within a country. In the countries included in our analysis, adoption rates tend to be higher for major grain crops and export crops than for root crops, reflecting either their higher commercial value or the effects of fertilizer subsidy programs, which typically target key staples and export crops. In Ethiopia, for example, fertilizer adoption rates for maize, wheat, and teff are around 90 percent, whereas the adoption rate for sorghum and millet is only around 30 percent. Variations in fertilizer use across countries and crops imply that the impacts of higher fertilizer prices will also vary across countries.

In addition to the direct impact of higher fertilizer prices on production costs, we model an additional impact channel where changes in fertilizer quantities affect productivity (or crop yields) directly. Since the precise quantities of fertilizer applied to crops and yield responses to changes in fertilization rates are difficult to estimate, especially for smaller crops or on plots that are intercropped, we adopt a conservative set of assumptions to estimate potential yield losses associated with the rise in fertilizer prices. First, we assume a price elasticity of farmers’ demand for fertilizer of −0.15, implying that a doubling of fertilizer prices leads to a 15 percent decline in fertilizer use. Despite a dearth of evidence on fertilizer own-price elasticities in developing countries, our assumed elasticity falls within the range estimated by Li et al. (2011) for South and East Asia, including wheat (−0.17), rice (−0.16), and maize (−0.11). Second, we assume crop yields on plots that are fertilized are 20 percent higher than on unfertilized plots. Our assumption is broadly consistent with the literature, which generally finds higher yields for farmers using fertilizer (see Hemming et al., 2018, in which authors found fertilizer and seed subsidies are associated with greater use of inputs, higher agricultural yields, and increased farmer incomes). Given crop-specific estimates of the share of land that is fertilized, we can estimate the change in fertilizer use and yields on fertilized plots as well as the average productivity change in each agricultural subsector under the assumption that yields on unfertilized plots are unaffected.

A key variable in this equation is the fertilizer price change. While in the initial simulation shock the international (real) price for fertilizer is doubled for all countries (Fig. 1), the change in the domestic price of fertilizer differs by country depending on whether fertilizer is also produced domestically. Domestically produced fertilizer accounts for a small amount of fertilizer supply in most countries. Zambia, for instance, relies entirely on imported fertilizer, and hence the domestic price of fertilizer also doubles. In Egypt, on the other hand, the country exports one-third of domestically produced fertilizer and imports about six percent of high-quality fertilizer supply, resulting in domestic fertilizer prices rising by only 27 percent, on average.

### 2.4 Model simulations

Several scenarios are designed to simulate the effects of higher world prices (see Fig. 1) and productivity losses from reduced fertilizer use (as discussed above). These are: (i) food price shocks: rising import and export prices for maize, wheat, and edible oils, and declining import and export prices for rice; (ii) fuel price shocks: rising import and export prices for crude oil and oil products; and (iii) fertilizer price shocks: rising fertilizer import prices combined with lower crop yields due to the resulting reduction in fertilizer use. The combined effect of all three impact channels is also simulated. Simulation results apply to the 2022 growing season, and so the results should be interpreted as reflecting the period after the initial direct and indirect (spillover) effects across sectors and households have occurred, but before governments introduce any mitigative policies, or the private sector adjusts its investments in response to the crisis. Impacts on economies and food systems are discussed first, before turning to impacts on poverty and food security.

## 3 Estimated impacts on economies and agrifood systems

**Fig. 2:**
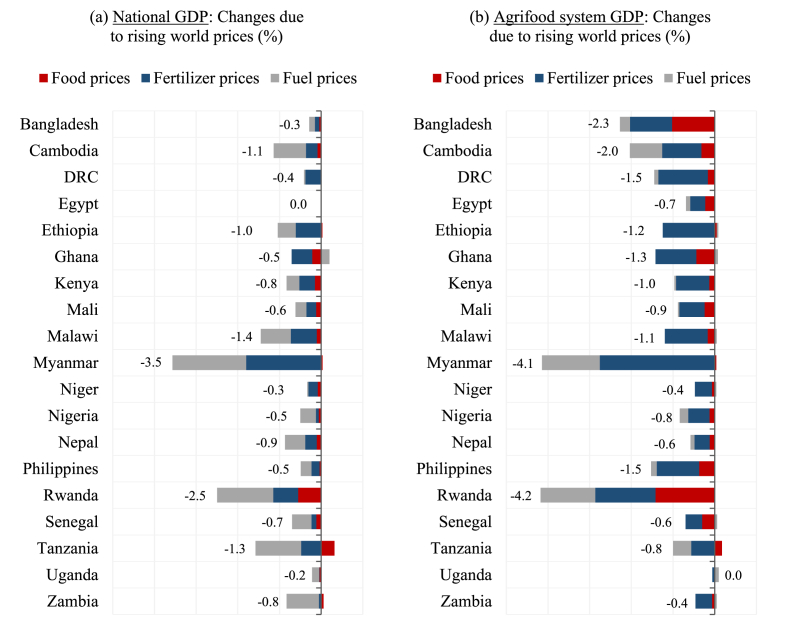
Estimated impacts on national and agrifood system GDP. Note: Agrifood system GDP includes primary agriculture, agro-processing, food services, agrifood related trade and transport, and domestic production of inputs into the primary agricultural and agro-processing sectors.

Source: Simulation results from IFPRI's RIAPA models (see www.ifpri.org/project/riapa-model) calibrated to data from IFPRI's Nexus Social Accounting Matrices (available at dataverse.harvard.edu/dataverse/sam).

Model results indicate that the impact of higher world prices on national GDP varies across countries but is generally modest. Panel A in Fig. 2 shows the estimated changes in GDP associated with the three impact channels, i.e., rising world prices for foods, fertilizers, and fuels. Overall, real GDP falls by less than one percent in 13 of the 19 countries, and has a negligible impact in Egypt, which is an exporter of natural gas, refined petroleum products, and fertilizers (i.e., for net-exporters, rising world prices represent windfall gains). In contrast, the largest GDP losses occur in Rwanda and Myanmar, which are agrarian economies for whom export prices did not rise and national production is highly reliant on imported inputs. On average, across the 19 countries, the world fuel price increase is the single largest contributor to national GDP losses (grey bars in the figure), followed by higher fertilizer prices and their associated productivity shocks (blue bars). Higher fuel prices cause production costs to rise in almost all sectors, because most products, including inputs, require transporting and thus indirectly use fuels. This results in higher producer prices and an across-the-board decline in demand for domestically produced goods.

Rising food prices have only a minimal impact on national GDP. On average, food price shocks cause less than a 0.1 percentage point decline in GDP. In fact, rising food prices contribute positively to GDP in countries that export maize, wheat, or oilseeds, and where agricultural exports are an important source of foreign exchange earnings (e.g., Tanzania and Zambia). Rising fuel and fertilizer prices cause agricultural production costs to increase and productivity to decline, and this contributes indirectly to rising domestic food prices. Thus, while the direct impact of global food price shocks is limited, the combined effect of global food, fuel, and fertilizer price increases on domestic food prices is significant. We revisit food prices below when discussing impacts on food security.

Panel B in Fig. 2 reports GDP changes in the agrifood system, which includes primary agriculture, agroprocessing, and agrifood-related trade, transport, and other services (Thurlow et al. *forthcoming*). With a few exceptions, the decline in agrifood system GDP is generally larger, in relative terms, than the decline in total GDP. Ten countries experience declines in agrifood system GDP of more than one percent, whereas only six countries see total GDP fall by more than one percent. Again, the greater exposure or vulnerability of the agrifood system is primarily due to the global crisis' concentrated impact on a narrow set of food and fertilizer commodities.

The three impact channels – world food, fuel, and fertilizer prices – affect different parts of the agrifood system. Overall, the fertilizer shock is the most important driver of agrifood system GDP losses as it has the most direct impact on primary agricultural production costs and productivity. This in turn leads to disruptions in downstream agrifood supply chains. Food price shocks have a more direct and disproportionate impact on agroprocessing because they raise the cost of imported inputs (e.g., domestic milling of wheat grain). Fuel price shocks, on the other hand, typically have a disproportionate effect on the food transport sector. Primary agriculture is also directly affected by rising fuel and food prices. For instance, higher food prices can benefit primary agriculture as consumers switch to locally sourced agrifood products. Conversely, in countries where agriculture is an intensive user of fuel-powered tillers, tractors, or irrigation, fuel prices raise production costs on the farm.

In summary, the economic impacts of the war in Ukraine and the ensuing global crisis vary widely across countries and depend crucially on country-specific production and trade structures. This cautions against generalizations about the effects of the crisis on developing countries as a group. That said, our results reveal the particularly adverse effects of the crisis on countries' agrifood systems, thus raising concerns about how the crisis will affect the world's poor and food insecure populations.

## 4 Estimated impacts on poverty and food security

### 4.1 Consumption spending and poverty

Real consumption spending, which includes the value of home consumption, falls in all 19 countries. The percentage decline in consumption is larger than that of GDP. In the modeling approach, households, rather than governments and investors, are viewed as likely to be more directly impacted. This approach is taken because many households are adversely affected by *both* rising prices and falling incomes. Food expenditure also accounts for a much larger share of household consumption than food production does in national GDP. As a result, rising food prices are relatively more important in explaining declines in consumption than they are in explaining GDP losses. Ultimately, while the global crisis does not substantially reduce national incomes (i.e., GDP), it does have an uneven impact on the macroeconomic distribution of national income, with consumption taking the brunt of the impact.

**Fig. 3:**
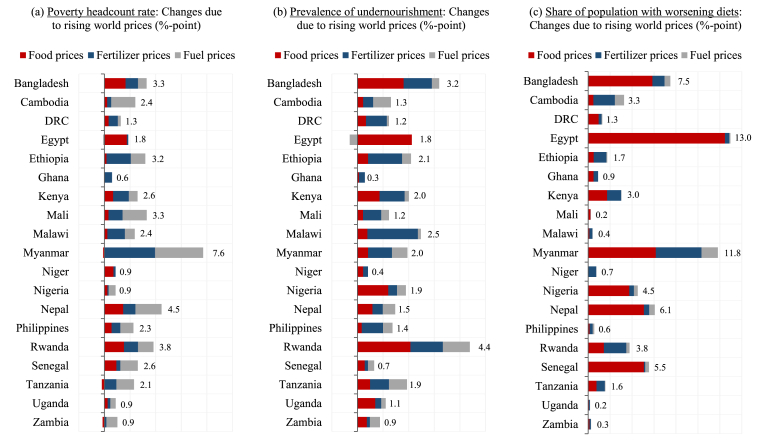
Estimated impacts on poverty and food security. Note: Poverty headcount rate is the population share with consumption below the US$1.90-a-day poverty line. Prevalence of undernourishment is the population share consuming less than the minimum calorie threshold. Diets are defined across six food groups and are deemed to have worsened if individuals become deprived in at least one additional group, where deprivation means consuming less than the minimum described by EAT-Lancet healthy reference diet.

Source: Simulation results from IFPRI's RIAPA models (see www.ifpri.org/project/riapa-model) calibrated to data from IFPRI's Nexus Social Accounting Matrices (available at dataverse.harvard.edu/dataverse/sam).

Falling household consumption leads to an increase in poverty. Our poverty assessment uses survey-based microsimulation models linked sequentially to economywide models (see Arndt et al., 2012). Each country model contains 15 representative household groups that separate the total population in rural farm, rural nonfarm, and urban households, each across expenditure quintiles. Every survey household is mapped to one of the models’ representative households. Estimated changes in consumption spending are transferred from the household in the model down to the household in the survey, where its poverty status is recomputed using the US$1.90-a-day international poverty line (Ferreira et al., 2015). Panel A in Fig. 3 indicates that the global price shocks increase national poverty headcount rates in all 19 countries. There is wide variation across countries, with poverty rates rising by as much as 7.6 percentage points in Myanmar, but by less than one percentage point in Ghana, Niger, Nigeria, Uganda, and Zambia.

**Fig. 4:**
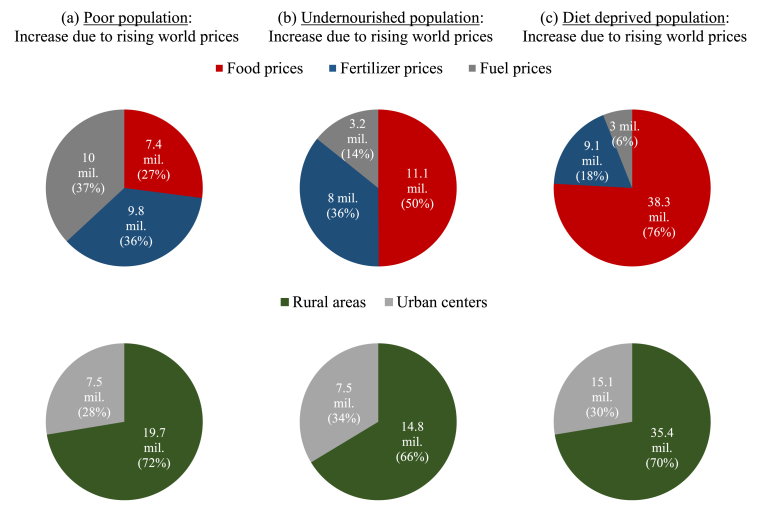
Decomposing increase in poor and food insecure population across the 19 countries. Note: Poor population share have consumption below the US$1.90-a-day poverty line. Undernourished population consumes less than the minimum calorie threshold. Diet deprived population become deprived in at least one additional food group (i.e., out of six groups and based on the EAT-Lancet heathy reference diet).

Source: Simulation results from IFPRI's RIAPA models (see www.ifpri.org/project/riapa-model) calibrated to data from IFPRI's Nexus Social Accounting Matrices (available at dataverse.harvard.edu/dataverse/sam).

The rise in national poverty equates to an additional 27.2 million people falling below the international poverty line across the 19 countries (see Table 1), with 72 percent of these people living in rural areas (see Panel A in Fig. 4). This reflects higher rural population shares and higher initial rural poverty rates. The increase in world fuel and fertilizer prices are the largest drivers of this increase in poverty, which is consistent with Fig. 2, which showed these impact channels driving the decline in national GDP or incomes. There is some variation across rural and urban areas, with rural poverty driven more by the fertilizer shocks, which reduces farm incomes, and urban poverty driven more by fuel shocks, which disproportionately affect the costs of commodities and services that urban households consume more intensively.

**Table 1:** Estimated increase in poor, undernourished, and diet deprived populations.

	Increased population (1000s)				Increased population (1000s)		
	Poor	Under-nourished	Diet deprived		Poor	Under-nourished	Diet deprived
**Total**	**27,190.4**	**22,261.3**	**50,434.4**	Myanmar	4,100.3	1,313.2	6,347.8
Bangladesh	5,077.9	5,288.1	11,700.4	Niger	208.7	97.3	177.7
Cambodia	389.9	214.0	533.1	Nigeria	1,782.6	3,905.6	8,350.0
DRC	1,118.3	1,048.1	1,158.8	Nepal	1,272.3	426.7	1,729.8
Egypt	1,832.1	1,900.8	13,510.4	Philippines	2,440.8	1,462.1	589.2
Ethiopia	3,567.9	2,308.5	1,915.3	Rwanda	484.9	555.2	476.3
Ghana	153.2	75.5	273.2	Senegal	418.6	108.0	880.8
Kenya	1,410.5	1,076.5	1,641.4	Tanzania	1,241.3	1,125.7	912.1
Mali	702.2	250.4	48.7	Uganda	388.0	492.0	72.3
Malawi	442.2	456.4	72.1	Zambia	158.8	157.2	44.9

Note: Poor population share have consumption below the US$1.90-a-day poverty line. Undernourished population consumes less than the minimum calorie threshold. Diet deprived population become deprived in at least one additional food group (i.e., out of six groups and based on the EAT-Lancet heathy reference diet).

Particularly after some time to adjust, smallholder farmers, who make up much of the rural population in Africa and Asia, may benefit from higher food prices, since this has the potential to raise farm incomes (see Headey and Martin 2016) and stimulate production with spillover implications for rural wages. In the analysis here, the net effect is typically negative for rural households. The models take into account the limited scope for adjustment of the farm sector in the time frame considered, nonfarm income sources of rural households, and the larger share of food in rural consumption baskets than farm incomes in total income.

### 4.2 Undernourishment and diet deprivation

Undernourishment also becomes more pervasive. A person is deemed undernourished when they consume fewer calories than what is required for a healthy life (FAO 2022). Calorie availability is assessed at household-level and requirements are adjusted for the age and gender composition of the household. With falling household consumption and rising poverty, the prevalence of undernourishment rises in all 19 countries, with increases in the range of 0.3–4.4 percentage points (see Panel B in Fig. 3). In total, 22.3 million more people become undernourished due to the global price crisis (see Table 1). As with poverty, most people who become undernourished (66 percent) live in rural areas (see Panel B in Fig. 4). Rising food prices are the most important driver of rising undernourishment – far more so than they were in explaining the increase in poverty. This is because rising prices for wheat and edible oils have a more direct and adverse impact on the cost and affordability of calorie heavy foods than they do on the broader consumer basket that determines a household's poverty status. Higher fertilizer prices are again an important driver of rising undernourishment. Our findings suggest that it is the concentration of world price increases across a narrow set of commodities important for people living near the calorie threshold, such as wheat and fertilizers, that amplifies the impacts of this crisis on food security indicators.

Our estimated changes in undernourishment may be somewhat overstated, because the demand system included the model – a Linear Expenditure System (LES) – incorporates minimum subsistence consumption quantities, which, in this context, limits substitution away from food items that become very expensive after the world price shock (i.e., wheat and edible oils). That said, declines in calorie availability is a common feature of negative economic shocks. For example, using longitudinal data from 52 countries, Headey and Ruel (2022) associate a ten percent decline in per capita income with a 15–18 percent increase in moderate or severe wasting in children under five.

Diet quality also deteriorates for many households. Diet quality is another important indicator of households' food and nutrition security status. The diet quality indicator used in this analysis, the Reference Diet Deprivation (ReDD) index (Pauw et al., 2021), is based on whether a household's food consumption meets the recommended calorie intakes across a diverse set of six food groups. Specifically, ReDD is a composite indicator of the incidence, breadth, and depth of diet deprivation within a population group or subgroup. The reference calorie intakes are defined by the healthy reference diet developed by the EAT-Lancet Commission (Willett et al., 2019). The food groups include staples (cereals and roots), fruits, vegetables, dairy, protein foods (animal-sourced and plant-based) and added fats (which includes edible oils). As with the poverty assessment, a survey-based microsimulation tool measures changes in the number of people who are diet deprived.

Prior to the crisis, few households accessed a healthy diet, with most failing to consume enough calories from food groups such as vegetables, dairy, and protein foods. Many households also consumed more than the recommended number of calories from staple foods. Households may therefore be deemed to have poor quality diets even if they are not undernourished in terms of energy intake. In Ghana, for example, our ReDD results reveal that the average person suffers four deprivations out of the six required food groups. Diets might deteriorate for several reasons. First, a decline in disposable incomes makes healthy diets less affordable. Second, an increase in the relative cost of food negatively affects the affordability of a healthy diet. Finally, relative changes in the average prices of different food groups may cause changes in food consumption patterns. As consumers shift consumption away from more expensive food groups and toward relatively cheaper ones, the rates of diet deprivation can change within the respective food groups. The net effect on diet quality depends on the magnitude of these consumption shifts relative to the calorie thresholds.

As discussed above, household disposable incomes decline across all 19 countries. Model results indicates that the cost of a healthy diet also increases in real terms in most countries, with the largest increase in the Philippines (4.7 percent). Exceptions include Bangladesh, Mali, Myanmar, and Nepal, where real diet costs decline slightly (i.e., food prices decline relative to nonfood prices). The largest decline is in Myanmar, where the real cost of a healthy diet falls by 2.4 percent. The cost of the “added fats” food group increases in all countries due to rising edible oil import prices. This is an important driver of the increase in the overall cost of the healthy reference diet. There is no readily observable consistency across countries in terms of movements in the cost of other food groups.

The combined effect of declining disposable incomes and rising diet costs (in most countries) causes diet quality to deteriorate. Relative food price changes, in turn, further encourage shifts in food consumption patterns, often exacerbating the quality of diets. Panel C in Fig. 3 reports the changing share of the population in each country who have diets that are worsening, i.e., the population that becomes deprived in at least one additional food group because of the crisis. Across the 19 studies, an additional 50.4 million people experience worsening diets, with 70 percent living in rural areas. Rising global food prices are overwhelmingly the driver of deteriorating diets, explaining 76 percent of the increase in the population with worsening diets. The increase in world fuel prices is a relatively minor driver of falling diet quality, mainly because the effect of this shock is diffused across the entire economy, including non-food sectors. In general, though, the nature of the 2022 global crisis makes it particularly detrimental for food security, especially diet quality.

The welfare metrics presented above for poverty, undernourishment, and diet quality are all threshold measures. The focus is on the increase in the number of people who fail to meet expenditure, calorie consumption, and/or dietary diversity thresholds. It merits mentioning that the welfare of people already poor, undernourished, and/or consuming low-quality diets declines as well, potentially dramatically as these people often live near survival thresholds. For example, Headey and Ruel (2022) find that food price inflation drives up wasting rates among children under five years old.

## 5 Summary and conclusion

Global food, fuel, and fertilizer prices rose rapidly in late-2021 and the first half 2022. This raised concerns about how these price shocks affect global food systems, poverty, and food security. We used economywide models to simulate the impacts of the global crisis on the economies and populations of 19 developing countries from the perspective of the information set available in late April or early May 2022. The models allowed us to trace the direct and indirect effects of these world price shocks on domestic economies, taking account of structural features and characteristics of those economies that ultimately determine the severity of the shock and help explain differences in impacts across countries. Important characteristics driving our results include, for example, the share of imports in total product supply; the importance of affected sectors and commodities for employment, income, and consumption; and farmers’ responses to rising fertilizer prices and the knock-on effect this may have in future agricultural cropping seasons. The models allow us to account for these characteristics and estimate the overall or net effect of the crisis at national and household scales within a coherent macroeconomic framework.

Although we find that there is wide variation in impacts across countries, there are general findings that can be drawn from across the case studies. National GDP losses, for example, are usually much smaller than GDP losses in the agrifood system. Moreover, it is rising fuel prices and fertilizer shocks that are the most important drivers of GDP losses, with rising food prices playing a less important role. This reflects the fact that wheat and edible oils, with some exceptions, are not typically important items in households’ consumption baskets in the countries assessed. In fact, in some countries, particularly those that export maize, wheat, oilseeds, and other agricultural products, rural farmers who are substantial net sellers of food may benefit from higher commodity prices, although the net effect on welfare for rural households as a group is consistently negative once we also account for the effects of higher fertilizer prices, reduced fertilizer use, and lower agricultural productivity.

We also find that household consumption falls in all 19 countries, including those that benefit modestly from exporting natural gas and crude oil. In contrast to what was observed for GDP, rising food prices are an important driver of consumption impacts in most countries. Rural populations are also adversely affected by fertilizer shocks, which directly impact agricultural productivity and rural incomes. Fuel prices, on the other hand, have a relatively stronger adverse impact on nonagricultural sectors and urban households’ consumption.

Falling household consumption leads to greater poverty in all countries, with a total of 27.2 million additional people pushed into poverty by the global crisis across the 19 countries included in the analysis. The majority of those that fall into poverty live in rural areas, although urban poor populations are also impacted. Consistent with the consumption result, rising food prices are an important driver of rising poverty in both rural and urban areas. Similar impacts are found for food security. An additional 22.3 million people become undernourished, i.e., they fail to obtain sufficient calories, mainly due to rising food prices. Moreover, 50.4 million people – approximately 4 percent of the total population across all 19 countries – become deprived in at least one additional food group, which is interpreted as a deterioration in diet quality.

Overall, our analysis confirms the significant adverse impact that the Russia-Ukraine war and ensuing global crisis has had on poverty and food security in developing countries. Despite heterogenous impacts, a cross-country comparison of results indicates that food systems and food insecure populations were particularly vulnerable to the 2022 global crisis. This is mainly because the crisis affected a narrow set of commodities that are particularly important for poor households, including the fertilizers that underpin farm incomes and food availability.

Fortunately, the sharp increase in commodity prices since mid-2021 had started to reverse by mid-2022. Falling food prices means that our estimated impacts, while relevant in mid-2022, may be overstated under the conditions prevailing later in the year. Our results suggest that the decline in palm oil prices may have significantly reduced the adverse effects of the crisis on diet quality, which was mainly driven by the higher cost of edible oils. However, fertilizer prices have remained well above mid-2021 levels, and so it is possible that impacts on poverty and undernourishment may persist into 2023.

Our analysis does not take into consideration any government interventions designed to mitigate the effects of the global shocks as well as the longer-term investments and adjustments that firms and households may undertake. Further analysis is needed on the mitigating effects of different policy and investment options, such as policies designed to lower costs or raise efficiency of fertilizer use; tax policies to offset higher import prices; and cash transfers to smooth consumption losses for the poor.

Finally, models and their mode of application can always be improved. Future analysis is also needed to confront the simulation results obtained here with evidence on actual behavior and outcomes on the ground. Overall, there remains considerable scope to better understand the implications of big shocks in a *timely* manner such that appropriate policy responses can be designed and implemented. Nevertheless, our analysis does underscore the benefits of mitigating policies to help offset the negative impacts on poverty and food insecurity of the global crisis.

## Declaration of competing interest

The authors declare that they have no known competing financial interests or personal relationships that could have appeared to influence the work reported in this paper.

## Data Availability

Data will be made available on request.

## Footnotes

1 Five Asian countries (Bangladesh, Cambodia, Myanmar, Nepal, Philippines) and 14 African countries (Democratic Republic of Congo, Egypt, Ethiopia, Ghana, Kenya, Mali, Malawi, Niger, Nigeria, Rwanda, Senegal, Tanzania, Uganda, Zambia).

2 The models use constant elasticity of substitution (CES) and transformation (CET) functions that allow imperfect substitution between domestic, imported and exported products/markets. Tables S1–S19 in the Supplementary Materials summarize base-year production and trade patterns for the 19 countries, and Table S20 provides the elasticity values used in the CES and CET functions. Details on model closures are also provided in the Supplementary Materials.

3 Fertilizer Use by Crop (FUBC) estimates for each country are shown in Table S21 in the Supplementary Materials.
