# Supplementary material for: The Ukraine war and rising commodity prices: Implications for developing countries
Source: Glob Food Sec. 2023 Mar;36:100680. doi: 10.1016/j.gfs.2023.100680 (PMC10015268; doi:10.1016/j.gfs.2023.100680)
Supplement: Multimedia component 1 [file mmc1.docx]

**Supplementary Materials**

*Macroeconomic and Factor Market Closure Rules*

The models include various mechanisms that determine how each economy adjusts in response to shocks in world prices and fertilizer use. Section 2.1, for example, describes product market mechanisms that determine domestic prices and bring about equilibrium in product demand and supply. The elasticities determining the ease at which consumers (producers) are able or willing to switch between domestic and foreign commodities (markets) are shown in the tables below. There are also other mechanisms that bring about equilibrium for within factor markets and the macroeconomy. We make a series of assumptions – called “closure rules” – that determine how these factor market and macroeconomic mechanisms operate in the models. These assumptions are described below.

Factor markets govern demand and supply of land, labor, and capital, and determine rental rates, wages, profit rates, respectively. Given the rapid onset of the global price crisis, we assume that agricultural land allocations are fixed by subsector. This means that farmers cannot adjust land allocations in response to changing prices, such as reallocating more land to wheat when wheat prices rise. In contrast, we assume labor can move between sectors in response to changes in labor demand and wage rates. Labor is disaggregated by rural/urban location and education level. We assume upward-sloping labor supply curves, which means that total employment falls if labor demand and wages decline, with the elasticity of labor supply being higher (less inelastic) for less-educated workers. Finally, capital is disaggregated based on the sector where it is used (i.e., crop, livestock, mining, and other sectors). We assume capital is sector specific or immobile. This means that capital/machinery cannot be easily repurposed or moved from, for example, mining to farming or financial services to petroleum/fertilizer production. Overall, our factor market closure rules are consistent with a model that aims to capture the immediate impacts of a rapid-onset shock like a sharp increase in world prices.

Closure rules also govern three macroeconomic accounts: current account balance, government fiscal balance, and saving-investment balance. First, we assume that the nominal exchange rate and current account balance (foreign capital inflows) are fixed, with both the domestic (producer) and consumer price indices adjusting endogenously within the models. This is consistent with countries’ Central Banks attempting to maintain nominal exchange rates over the short-term in response to shocks, and with the difficulties that countries face in increasing foreign borrowing to cover higher import costs. Second, our impact analysis does not account for possible government policy responses to the global price shocks. As such, government recurrent spending and tax rates are fixed, with revenues adjusting in response to changes in commodity trade/prices and household incomes. Finally, we assume that savings rates are fixed so that total private savings is primarily determined by income levels, and that total available savings (loanable funds), which also includes endogenous government savings and exogenous foreign savings (capital inflows), determines the total level of investment demand in the economy (i.e., a savings-driven investment closure). These three sets of macro closure rules are standard for short-term CGE modeling analysis, i.e., they reflect the immediate foreign exchange and borrowing constraints that countries and governments faced in the wake of the global crisis.

Table S1: Bangladesh Production and Trade Structure

|  | Share of total value (%) | | | Export-intensity (%) | Import-intensity (%) |
| --- | --- | --- | --- | --- | --- |
|  | GDP | Exports | Imports |  |  |
|  |  |  |  |  |  |
| All sectors | 100.0 | 100.0 | 100.0 | 7.7 | 11.8 |
|  |  |  |  |  |  |
| Agriculture | 13.5 | 0.6 | 9.7 | 0.4 | 9.1 |
| Crops | 7.2 | 0.6 | 9.5 | 0.9 | 18.1 |
| *of which* Maize | 0.2 | 0.0 | 0.2 | 0.0 | 13.5 |
| Sorghum and millet | 0.0 | 0.0 | 0.0 | 0.0 | 0.0 |
| Rice | 3.8 | 0.0 | 0.0 | 0.0 | 0.0 |
| Wheat and barley | 0.1 | 0.0 | 2.4 | 0.0 | 79.4 |
| Other oilseeds | 0.3 | 0.0 | 1.3 | 4.3 | 71.1 |
| Livestock | 1.8 | 0.0 | 0.1 | 0.0 | 0.6 |
| Other agriculture | 4.4 | 0.0 | 0.2 | 0.0 | 0.6 |
|  |  |  |  |  |  |
| Mining | 1.8 | 0.0 | 2.6 | 0.1 | 15.1 |
| *of which* Crude oil | 0.0 | 0.0 | 0.8 | 22.4 | 96.3 |
| Natural gas | 0.6 | 0.0 | 0.0 | 0.0 | 0.0 |
|  |  |  |  |  |  |
| Manufacturing | 19.5 | 90.3 | 72.8 | 21.7 | 25.9 |
| *of which* Agro-processing | 2.8 | 2.5 | 13.4 | 3.2 | 18.8 |
| *of which* Maize milling | 0.0 | 0.0 | 0.0 | 0.0 | 0.0 |
| Sorghum & millet milling | 0.0 | 0.0 | 0.0 | 0.0 | 0.0 |
| Rice milling | 0.4 | 0.0 | 0.8 | 0.0 | 4.0 |
| Wheat & barley milling | 0.1 | 0.0 | 0.0 | 0.0 | 0.0 |
| Fats and oils | 0.1 | 0.0 | 0.5 | 0.0 | 35.1 |
| Other manufacturing | 16.7 | 87.8 | 59.3 | 26.1 | 28.0 |
| Petroleum | 0.1 | 0.4 | 9.1 | 36.4 | 92.9 |
| Fertilizers | 0.1 | 0.0 | 2.3 | 2.1 | 72.6 |
|  |  |  |  |  |  |
| Other industry | 9.5 | 0.0 | 0.0 | 0.0 | 0.0 |
|  |  |  |  |  |  |
| Services | 55.6 | 9.1 | 14.9 | 2.0 | 4.0 |
| Trade and hotels | 14.5 | 0.5 | 1.2 | 0.4 | 1.2 |
| Transport and communication | 9.3 | 1.3 | 9.8 | 1.4 | 11.9 |
| Finance and business services | 11.7 | 2.8 | 3.4 | 3.0 | 4.4 |
| Government services | 9.4 | 4.5 | 0.5 | 5.3 | 0.8 |
| Other services | 10.7 | 0.0 | 0.0 | 0.0 | 0.0 |
|  |  |  |  |  |  |

Source: Aggregated base-year sector and product data from RIAPA’s Nexus SAMs.

Note: Export-intensity is the share of exports in total output; and import-intensity is share of imports in total demand. GDP is total gross domestic product.

Table S2: DRC Production and Trade Structure

|  | Share of total value (%) | | | Export-intensity (%) | Import-intensity (%) |
| --- | --- | --- | --- | --- | --- |
|  | GDP | Exports | Imports |  |  |
|  |  |  |  |  |  |
| All sectors | 100.0 | 100.0 | 100.0 | 19.4 | 24.3 |
|  |  |  |  |  |  |
| Agriculture | 20.1 | 0.6 | 0.5 | 0.6 | 0.8 |
| Crops | 19.5 | 0.2 | 0.4 | 0.2 | 0.6 |
| *of which* Maize | 6.3 | 0.0 | 0.0 | 0.0 | 0.0 |
| Sorghum and millet | 0.1 | 0.0 | 0.0 | 0.0 | 0.0 |
| Rice | 7.2 | 0.0 | 0.0 | 0.0 | 0.0 |
| Wheat and barley | 0.0 | 0.0 | 0.3 | 0.0 | 96.4 |
| Other oilseeds | 0.1 | 0.0 | 0.0 | 0.0 | 0.0 |
| Livestock | 0.0 | 0.0 | 0.2 | 0.0 | 54.7 |
| Other agriculture | 0.6 | 0.4 | 0.0 | 16.0 | 0.0 |
|  |  |  |  |  |  |
| Mining | 20.6 | 98.6 | 0.8 | 96.6 | 21.6 |
| *of which* Crude oil | 1.0 | 6.5 | 0.0 | 100.0 | 0.0 |
| Natural gas | 0.0 | 0.0 | 0.0 | 0.0 | 0.0 |
|  |  |  |  |  |  |
| Manufacturing | 18.7 | 0.1 | 77.0 | 0.1 | 41.7 |
| *of which* Agro-processing | 15.9 | 0.0 | 12.9 | 0.0 | 11.1 |
| *of which* Maize milling | 3.2 | 0.0 | 0.0 | 0.0 | 0.0 |
| Sorghum & millet milling | 0.1 | 0.0 | 0.0 | 0.0 | 0.0 |
| Rice milling | 1.5 | 0.0 | 0.4 | 0.0 | 1.3 |
| Wheat & barley milling | 0.0 | 0.0 | 0.7 | 0.0 | 76.1 |
| Fats and oils | 0.0 | 0.0 | 0.2 | 0.0 | 100.0 |
| Other manufacturing | 2.8 | 0.1 | 64.1 | 0.5 | 83.3 |
| Petroleum | 0.0 | 0.0 | 2.4 | 0.0 | 100.0 |
| Fertilizers | 0.0 | 0.0 | 2.1 | 0.0 | 100.0 |
|  |  |  |  |  |  |
| Other industry | 7.1 | 0.0 | 0.2 | 0.0 | 0.6 |
|  |  |  |  |  |  |
| Services | 33.4 | 0.7 | 21.4 | 0.5 | 13.5 |
| Trade and hotels | 13.9 | 0.5 | 0.3 | 0.9 | 0.5 |
| Transport and communication | 8.6 | 0.0 | 12.9 | 0.0 | 27.9 |
| Finance and business services | 5.5 | 0.0 | 8.2 | 0.0 | 26.6 |
| Government services | 5.2 | 0.2 | 0.0 | 0.7 | 0.0 |
| Other services | 0.2 | 0.0 | 0.1 | 0.0 | 6.9 |
|  |  |  |  |  |  |

Source: Aggregated base-year sector and product data from RIAPA’s Nexus SAMs.

Note: Export-intensity is the share of exports in total output; and import-intensity is share of imports in total demand. GDP is total gross domestic product.

Table S3: Egypt Production and Trade Structure

|  | Share of total value (%) | | | Export-intensity (%) | Import-intensity (%) |
| --- | --- | --- | --- | --- | --- |
|  | GDP | Exports | Imports |  |  |
|  |  |  |  |  |  |
| All sectors | 100.0 | 100.0 | 100.0 | 8.7 | 14.4 |
|  |  |  |  |  |  |
| Agriculture | 12.5 | 4.6 | 8.7 | 3.5 | 12.0 |
| Crops | 6.2 | 4.6 | 8.4 | 6.8 | 20.7 |
| *of which* Maize | 1.0 | 0.0 | 2.0 | 0.0 | 31.6 |
| Sorghum and millet | 0.1 | 0.0 | 0.0 | 0.0 | 0.0 |
| Rice | 0.4 | 0.0 | 0.0 | 0.0 | 0.0 |
| Wheat and barley | 0.3 | 0.0 | 1.2 | 0.0 | 41.2 |
| Other oilseeds | 0.1 | 0.1 | 2.1 | 7.7 | 79.1 |
| Livestock | 5.1 | 0.0 | 0.2 | 0.1 | 0.6 |
| Other agriculture | 1.2 | 0.0 | 0.0 | 0.0 | 0.6 |
|  |  |  |  |  |  |
| Mining | 9.9 | 7.3 | 2.9 | 9.8 | 6.2 |
| *of which* Crude oil | 5.2 | 0.0 | 0.4 | 0.0 | 1.6 |
| Natural gas | 3.2 | 2.8 | 0.0 | 12.4 | 0.0 |
|  |  |  |  |  |  |
| Manufacturing | 18.2 | 44.5 | 64.6 | 11.3 | 25.2 |
| *of which* Agro-processing | 3.6 | 5.1 | 8.0 | 6.0 | 17.0 |
| *of which* Maize milling | 0.0 | 0.0 | 0.0 | 0.0 | 0.0 |
| Sorghum & millet milling | 0.0 | 0.0 | 0.0 | 0.0 | 0.0 |
| Rice milling | 0.0 | 0.0 | 0.3 | 0.0 | 32.2 |
| Wheat & barley milling | 0.1 | 0.4 | 0.0 | 17.0 | 0.0 |
| Fats and oils | 0.3 | 0.6 | 0.6 | 5.0 | 9.6 |
| Other manufacturing | 14.6 | 39.4 | 56.6 | 12.7 | 27.3 |
| Petroleum | 4.1 | 9.9 | 7.6 | 11.6 | 15.0 |
| Fertilizers | 0.3 | 2.7 | 0.2 | 30.7 | 6.0 |
|  |  |  |  |  |  |
| Other industry | 7.9 | 0.0 | 0.0 | 0.0 | 0.0 |
|  |  |  |  |  |  |
| Services | 51.6 | 43.6 | 23.8 | 9.9 | 8.6 |
| Trade and hotels | 15.8 | 18.3 | 0.0 | 13.9 | 0.0 |
| Transport and communication | 9.5 | 18.8 | 11.9 | 22.8 | 22.8 |
| Finance and business services | 14.3 | 4.4 | 10.7 | 3.5 | 12.3 |
| Government services | 9.7 | 1.8 | 1.1 | 2.2 | 2.0 |
| Other services | 2.3 | 0.3 | 0.2 | 1.4 | 1.2 |
|  |  |  |  |  |  |

Source: Aggregated base-year sector and product data from RIAPA’s Nexus SAMs.

Note: Export-intensity is the share of exports in total output; and import-intensity is share of imports in total demand. GDP is total gross domestic product.

Table S4: Ethiopia Production and Trade Structure

|  | Share of total value (%) | | | Export-intensity (%) | Import-intensity (%) |
| --- | --- | --- | --- | --- | --- |
|  | GDP | Exports | Imports |  |  |
|  |  |  |  |  |  |
| All sectors | 100.0 | 100.0 | 100.0 | 4.0 | 13.6 |
|  |  |  |  |  |  |
| Agriculture | 33.1 | 30.6 | 2.6 | 4.8 | 1.8 |
| Crops | 21.7 | 29.9 | 2.5 | 8.2 | 3.2 |
| *of which* Maize | 2.2 | 0.0 | 0.2 | 0.0 | 1.5 |
| Sorghum and millet | 3.0 | 0.0 | 0.2 | 0.0 | 2.3 |
| Rice | 0.1 | 0.0 | 0.0 | 0.0 | 0.1 |
| Wheat and barley | 3.6 | 0.0 | 1.7 | 0.0 | 7.9 |
| Other oilseeds | 0.4 | 5.7 | 0.0 | 84.4 | 0.0 |
| Livestock | 8.3 | 0.7 | 0.0 | 0.5 | 0.1 |
| Other agriculture | 3.1 | 0.0 | 0.0 | 0.0 | 0.0 |
|  |  |  |  |  |  |
| Mining | 0.2 | 0.0 | 0.4 | 0.0 | 9.5 |
| *of which* Crude oil | 0.0 | 0.0 | 0.0 | 0.0 | 0.0 |
| Natural gas | 0.0 | 0.0 | 0.0 | 0.0 | 0.0 |
|  |  |  |  |  |  |
| Manufacturing | 6.0 | 8.2 | 66.9 | 3.2 | 50.1 |
| *of which* Agro-processing | 2.7 | 2.0 | 4.7 | 1.4 | 11.4 |
| *of which* Maize milling | 0.1 | 0.0 | 0.0 | 0.0 | 0.0 |
| Sorghum & millet milling | 0.0 | 0.0 | 0.0 | 0.0 | 0.0 |
| Rice milling | 0.0 | 0.0 | 0.8 | 0.0 | 88.9 |
| Wheat & barley milling | 0.0 | 0.0 | 0.2 | 0.0 | 3.5 |
| Fats and oils | 0.1 | 0.0 | 0.1 | 0.0 | 6.8 |
| Other manufacturing | 3.3 | 6.1 | 62.2 | 5.8 | 68.8 |
| Petroleum | 0.0 | 0.0 | 11.1 | 0.0 | 99.4 |
| Fertilizers | 0.0 | 0.0 | 3.0 | 0.0 | 98.4 |
|  |  |  |  |  |  |
| Other industry | 23.0 | 0.0 | 2.4 | 0.0 | 1.2 |
|  |  |  |  |  |  |
| Services | 37.7 | 61.2 | 27.7 | 6.5 | 8.3 |
| Trade and hotels | 15.4 | 3.2 | 0.2 | 1.0 | 0.2 |
| Transport and communication | 4.5 | 50.9 | 21.9 | 26.6 | 31.0 |
| Finance and business services | 7.1 | 4.2 | 5.2 | 2.0 | 6.8 |
| Government services | 8.2 | 2.9 | 0.5 | 1.7 | 0.8 |
| Other services | 2.4 | 0.0 | 0.0 | 0.0 | 0.0 |
|  |  |  |  |  |  |

Source: Aggregated base-year sector and product data from RIAPA’s Nexus SAMs.

Note: Export-intensity is the share of exports in total output; and import-intensity is share of imports in total demand. GDP is total gross domestic product.

Table S5: Ghana Production and Trade Structure

|  | Share of total value (%) | | | Export-intensity (%) | Import-intensity (%) |
| --- | --- | --- | --- | --- | --- |
|  | GDP | Exports | Imports |  |  |
|  |  |  |  |  |  |
| All sectors | 100.0 | 100.0 | 100.0 | 15.3 | 19.0 |
|  |  |  |  |  |  |
| Agriculture | 18.2 | 10.9 | 1.3 | 12.0 | 2.4 |
| Crops | 14.5 | 10.9 | 1.2 | 18.3 | 3.4 |
| *of which* Maize | 1.0 | 0.0 | 0.1 | 0.0 | 1.9 |
| Sorghum and millet | 0.3 | 0.0 | 0.0 | 0.0 | 0.0 |
| Rice | 0.6 | 0.0 | 0.0 | 0.0 | 0.0 |
| Wheat and barley | 0.0 | 0.0 | 0.9 | 0.0 | 100.0 |
| Other oilseeds | 0.8 | 0.2 | 0.0 | 4.7 | 0.0 |
| Livestock | 1.6 | 0.0 | 0.1 | 0.0 | 1.1 |
| Other agriculture | 2.2 | 0.0 | 0.0 | 0.0 | 0.0 |
|  |  |  |  |  |  |
| Mining | 14.3 | 67.8 | 0.1 | 74.1 | 0.4 |
| *of which* Crude oil | 6.5 | 44.7 | 0.0 | 76.5 | 0.0 |
| Natural gas | 0.0 | 0.0 | 0.0 | 0.0 | 0.0 |
|  |  |  |  |  |  |
| Manufacturing | 10.9 | 6.9 | 66.5 | 5.0 | 40.4 |
| *of which* Agro-processing | 3.4 | 4.0 | 10.9 | 8.0 | 29.2 |
| *of which* Maize milling | 0.4 | 0.0 | 0.0 | 0.0 | 0.0 |
| Sorghum & millet milling | 0.2 | 0.0 | 0.0 | 0.0 | 0.0 |
| Rice milling | 0.4 | 0.0 | 2.6 | 0.0 | 45.9 |
| Wheat & barley milling | 0.1 | 0.0 | 0.0 | 0.0 | 0.0 |
| Fats and oils | 0.7 | 0.0 | 0.4 | 0.0 | 10.1 |
| Other manufacturing | 7.5 | 3.0 | 55.6 | 3.4 | 45.4 |
| Petroleum | 0.8 | 0.0 | 1.7 | 0.0 | 14.5 |
| Fertilizers | 0.0 | 0.0 | 1.4 | 3.5 | 81.9 |
|  |  |  |  |  |  |
| Other industry | 8.2 | 2.4 | 1.7 | 3.3 | 2.4 |
|  |  |  |  |  |  |
| Services | 48.4 | 11.9 | 30.5 | 4.7 | 11.7 |
| Trade and hotels | 19.9 | 7.3 | 0.0 | 7.2 | 0.0 |
| Transport and communication | 10.1 | 2.8 | 13.0 | 5.6 | 22.1 |
| Finance and business services | 8.0 | 0.0 | 5.2 | 0.0 | 11.4 |
| Government services | 9.3 | 0.0 | 11.8 | 0.0 | 19.3 |
| Other services | 1.0 | 1.7 | 0.6 | 21.7 | 9.0 |
|  |  |  |  |  |  |

Source: Aggregated base-year sector and product data from RIAPA’s Nexus SAMs.

Note: Export-intensity is the share of exports in total output; and import-intensity is share of imports in total demand. GDP is total gross domestic product.

Table S6: Kenya Production and Trade Structure

|  | Share of total value (%) | | | Export-intensity (%) | Import-intensity (%) |
| --- | --- | --- | --- | --- | --- |
|  | GDP | Exports | Imports |  |  |
|  |  |  |  |  |  |
| All sectors | 100.0 | 100.0 | 100.0 | 7.0 | 14.1 |
|  |  |  |  |  |  |
| Agriculture | 23.1 | 12.1 | 2.5 | 5.2 | 2.4 |
| Crops | 16.6 | 11.9 | 2.5 | 7.2 | 3.4 |
| *of which* Maize | 3.4 | 0.1 | 0.3 | 0.2 | 2.4 |
| Sorghum and millet | 0.2 | 0.2 | 0.2 | 10.3 | 20.9 |
| Rice | 0.1 | 0.0 | 0.0 | 0.0 | 0.3 |
| Wheat and barley | 0.3 | 0.2 | 1.3 | 6.7 | 54.2 |
| Other oilseeds | 0.0 | 0.0 | 0.0 | 7.3 | 10.0 |
| Livestock | 4.0 | 0.1 | 0.0 | 0.3 | 0.1 |
| Other agriculture | 2.5 | 0.0 | 0.0 | 0.0 | 0.0 |
|  |  |  |  |  |  |
| Mining | 0.8 | 1.6 | 0.5 | 13.9 | 8.8 |
| *of which* Crude oil | 0.0 | 0.0 | 0.1 | 0.0 | 99.1 |
| Natural gas | 0.0 | 0.0 | 0.0 | 0.0 | 0.0 |
|  |  |  |  |  |  |
| Manufacturing | 8.6 | 38.0 | 79.5 | 16.6 | 46.1 |
| *of which* Agro-processing | 5.0 | 22.9 | 7.6 | 16.9 | 12.7 |
| *of which* Maize milling | 0.1 | 0.0 | 0.0 | 0.0 | 0.7 |
| Sorghum & millet milling | 0.0 | 0.0 | 0.0 | 0.1 | 0.8 |
| Rice milling | 0.0 | 0.0 | 0.9 | 0.2 | 68.0 |
| Wheat & barley milling | 0.2 | 0.0 | 0.0 | 0.1 | 0.1 |
| Fats and oils | 0.4 | 0.0 | 0.7 | 0.0 | 15.8 |
| Other manufacturing | 3.6 | 15.1 | 72.0 | 16.3 | 65.3 |
| Petroleum | 0.0 | 0.0 | 10.1 | 0.0 | 82.3 |
| Fertilizers | 0.0 | 0.4 | 2.5 | 76.7 | 99.7 |
|  |  |  |  |  |  |
| Other industry | 8.9 | 0.0 | 2.0 | 0.0 | 2.5 |
|  |  |  |  |  |  |
| Services | 58.6 | 48.3 | 15.5 | 6.1 | 3.5 |
| Trade and hotels | 10.2 | 0.2 | 0.0 | 0.1 | 0.0 |
| Transport and communication | 15.5 | 21.0 | 6.2 | 9.1 | 5.0 |
| Finance and business services | 17.7 | 10.9 | 7.7 | 4.9 | 6.0 |
| Government services | 12.6 | 0.0 | 0.0 | 0.0 | 0.0 |
| Other services | 2.6 | 16.3 | 1.6 | 64.4 | 23.2 |
|  |  |  |  |  |  |

Source: Aggregated base-year sector and product data from RIAPA’s Nexus SAMs.

Note: Export-intensity is the share of exports in total output; and import-intensity is share of imports in total demand. GDP is total gross domestic product.

Table S7: Cambodia Production and Trade Structure

|  | Share of total value (%) | | | Export-intensity (%) | Import-intensity (%) |
| --- | --- | --- | --- | --- | --- |
|  | GDP | Exports | Imports |  |  |
|  |  |  |  |  |  |
| All sectors | 100.0 | 100.0 | 100.0 | 26.4 | 31.4 |
|  |  |  |  |  |  |
| Agriculture | 21.9 | 0.7 | 1.8 | 1.6 | 4.8 |
| Crops | 13.3 | 0.7 | 0.3 | 2.9 | 1.4 |
| *of which* Maize | 0.5 | 0.0 | 0.0 | 0.0 | 2.5 |
| Sorghum and millet | 0.0 | 0.0 | 0.0 | 0.0 | 0.0 |
| Rice | 8.8 | 0.0 | 0.0 | 0.1 | 0.0 |
| Wheat and barley | 0.0 | 0.0 | 0.1 | 0.0 | 97.6 |
| Other oilseeds | 0.1 | 0.0 | 0.0 | 2.0 | 2.8 |
| Livestock | 2.7 | 0.0 | 1.5 | 0.4 | 26.5 |
| Other agriculture | 6.0 | 0.0 | 0.0 | 0.0 | 0.0 |
|  |  |  |  |  |  |
| Mining | 1.9 | 5.3 | 0.0 | 97.2 | 25.3 |
| *of which* Crude oil | 0.0 | 0.0 | 0.0 | 0.0 | 0.0 |
| Natural gas | 0.0 | 0.0 | 0.0 | 0.0 | 99.6 |
|  |  |  |  |  |  |
| Manufacturing | 18.4 | 62.9 | 80.9 | 61.3 | 71.6 |
| *of which* Agro-processing | 5.9 | 3.3 | 7.7 | 13.5 | 32.3 |
| *of which* Maize milling | 0.0 | 0.0 | 0.0 | 0.0 | 42.2 |
| Sorghum & millet milling | 0.0 | 0.0 | 0.0 | 0.0 | 0.0 |
| Rice milling | 0.8 | 1.3 | 0.0 | 40.0 | 0.0 |
| Wheat & barley milling | 0.0 | 0.0 | 0.0 | 0.0 | 0.0 |
| Fats and oils | 0.0 | 0.0 | 0.2 | 0.0 | 95.3 |
| Other manufacturing | 12.5 | 59.6 | 73.2 | 76.2 | 81.6 |
| Petroleum | 0.0 | 0.0 | 11.7 | 0.0 | 97.5 |
| Fertilizers | 0.1 | 0.0 | 0.5 | 0.0 | 76.2 |
|  |  |  |  |  |  |
| Other industry | 15.1 | 0.0 | 0.7 | 0.0 | 1.4 |
|  |  |  |  |  |  |
| Services | 42.6 | 31.1 | 16.5 | 19.9 | 12.0 |
| Trade and hotels | 17.3 | 14.0 | 4.8 | 22.8 | 9.4 |
| Transport and communication | 9.8 | 14.4 | 8.7 | 41.0 | 30.3 |
| Finance and business services | 6.5 | 1.2 | 2.9 | 4.9 | 11.3 |
| Government services | 5.0 | 1.4 | 0.1 | 10.0 | 0.9 |
| Other services | 4.0 | 0.0 | 0.0 | 0.0 | 0.0 |
|  |  |  |  |  |  |

Source: Aggregated base-year sector and product data from RIAPA’s Nexus SAMs.

Note: Export-intensity is the share of exports in total output; and import-intensity is share of imports in total demand. GDP is total gross domestic product.

Table S8: Mali Production and Trade Structure

|  | Share of total value (%) | | | Export-intensity (%) | Import-intensity (%) |
| --- | --- | --- | --- | --- | --- |
|  | GDP | Exports | Imports |  |  |
|  |  |  |  |  |  |
| All sectors | 100.0 | 100.0 | 100.0 | 14.2 | 19.5 |
|  |  |  |  |  |  |
| Agriculture | 40.1 | 4.8 | 2.3 | 2.9 | 2.1 |
| Crops | 19.4 | 0.9 | 2.2 | 1.1 | 3.8 |
| *of which* Maize | 1.9 | 0.0 | 0.0 | 0.0 | 0.0 |
| Sorghum and millet | 4.1 | 0.0 | 0.0 | 0.0 | 0.0 |
| Rice | 3.8 | 0.0 | 0.0 | 0.0 | 0.0 |
| Wheat and barley | 0.0 | 0.0 | 1.3 | 0.0 | 100.0 |
| Other oilseeds | 0.9 | 0.7 | 0.0 | 13.8 | 0.0 |
| Livestock | 16.6 | 3.9 | 0.1 | 5.9 | 0.2 |
| Other agriculture | 4.1 | 0.0 | 0.0 | 0.0 | 0.0 |
|  |  |  |  |  |  |
| Mining | 0.6 | 0.0 | 0.2 | 0.0 | 2.8 |
| *of which* Crude oil | 0.0 | 0.0 | 0.0 | 0.0 | 0.0 |
| Natural gas | 0.0 | 0.0 | 0.0 | 0.0 | 0.0 |
|  |  |  |  |  |  |
| Manufacturing | 17.7 | 87.7 | 62.6 | 39.7 | 39.5 |
| *of which* Agro-processing | 4.0 | 0.4 | 6.0 | 0.6 | 12.2 |
| *of which* Maize milling | 0.0 | 0.0 | 0.0 | 0.0 | 0.0 |
| Sorghum & millet milling | 0.1 | 0.0 | 0.0 | 0.0 | 0.0 |
| Rice milling | 0.4 | 0.0 | 0.8 | 0.0 | 7.7 |
| Wheat & barley milling | 0.0 | 0.0 | 0.2 | 0.0 | 39.6 |
| Fats and oils | 0.4 | 0.0 | 0.5 | 0.0 | 10.5 |
| Other manufacturing | 13.7 | 87.3 | 56.6 | 56.9 | 54.6 |
| Petroleum | 0.0 | 0.0 | 18.2 | 0.0 | 94.9 |
| Fertilizers | 0.0 | 0.0 | 1.6 | 0.0 | 76.9 |
|  |  |  |  |  |  |
| Other industry | 5.2 | 0.0 | 0.7 | 0.0 | 1.8 |
|  |  |  |  |  |  |
| Services | 36.4 | 7.5 | 34.2 | 3.3 | 16.9 |
| Trade and hotels | 11.3 | 1.9 | 0.0 | 2.2 | 0.0 |
| Transport and communication | 5.2 | 0.6 | 9.5 | 1.6 | 25.2 |
| Finance and business services | 4.9 | 2.8 | 4.0 | 6.8 | 12.2 |
| Government services | 13.0 | 2.3 | 8.7 | 3.9 | 16.8 |
| Other services | 2.0 | 0.0 | 12.0 | 0.0 | 64.0 |
|  |  |  |  |  |  |

Source: Aggregated base-year sector and product data from RIAPA’s Nexus SAMs.

Note: Export-intensity is the share of exports in total output; and import-intensity is share of imports in total demand. GDP is total gross domestic product.

Table S9: Myanmar Production and Trade Structure

|  | Share of total value (%) | | | Export-intensity (%) | Import-intensity (%) |
| --- | --- | --- | --- | --- | --- |
|  | GDP | Exports | Imports |  |  |
|  |  |  |  |  |  |
| All sectors | 100.0 | 100.0 | 100.0 | 13.4 | 16.8 |
|  |  |  |  |  |  |
| Agriculture | 22.0 | 9.0 | 2.6 | 8.1 | 2.8 |
| Crops | 14.7 | 6.2 | 2.6 | 8.7 | 4.3 |
| *of which* Maize | 0.5 | 0.2 | 0.0 | 8.1 | 0.0 |
| Sorghum and millet | 0.2 | 0.0 | 0.0 | 0.0 | 0.0 |
| Rice | 4.7 | 0.0 | 0.0 | 0.0 | 0.0 |
| Wheat and barley | 0.0 | 0.0 | 2.6 | 0.0 | 91.5 |
| Other oilseeds | 1.0 | 0.6 | 0.0 | 15.6 | 0.0 |
| Livestock | 3.6 | 0.0 | 0.0 | 0.2 | 0.0 |
| Other agriculture | 3.7 | 2.8 | 0.0 | 22.4 | 0.0 |
|  |  |  |  |  |  |
| Mining | 3.3 | 7.4 | 0.4 | 44.7 | 4.2 |
| *of which* Crude oil | 0.0 | 0.0 | 0.4 | 0.0 | 100.0 |
| Natural gas | 0.6 | 2.6 | 0.0 | 69.9 | 0.0 |
|  |  |  |  |  |  |
| Manufacturing | 21.9 | 57.4 | 77.3 | 19.7 | 30.7 |
| *of which* Agro-processing | 8.2 | 11.0 | 6.9 | 8.5 | 6.6 |
| *of which* Maize milling | 0.2 | 0.0 | 0.0 | 0.0 | 0.0 |
| Sorghum & millet milling | 0.0 | 0.0 | 0.0 | 0.0 | 0.0 |
| Rice milling | 1.3 | 6.3 | 0.0 | 26.2 | 0.0 |
| Wheat & barley milling | 0.2 | 0.0 | 0.0 | 0.0 | 0.0 |
| Fats and oils | 0.2 | 0.0 | 0.3 | 0.0 | 11.9 |
| Other manufacturing | 13.7 | 46.4 | 70.4 | 28.5 | 45.0 |
| Petroleum | 0.1 | 0.0 | 16.5 | 0.0 | 93.5 |
| Fertilizers | 0.0 | 0.0 | 2.5 | 0.0 | 73.2 |
|  |  |  |  |  |  |
| Other industry | 8.5 | 2.1 | 1.7 | 2.8 | 2.3 |
|  |  |  |  |  |  |
| Services | 44.5 | 24.1 | 18.1 | 10.6 | 8.1 |
| Trade and hotels | 27.8 | 16.1 | 0.5 | 13.5 | 0.5 |
| Transport and communication | 7.8 | 5.1 | 11.9 | 10.8 | 22.2 |
| Finance and business services | 3.0 | 0.1 | 5.0 | 0.4 | 15.5 |
| Government services | 5.4 | 2.9 | 0.6 | 9.0 | 2.0 |
| Other services | 0.5 | 0.0 | 0.0 | 0.0 | 0.0 |
|  |  |  |  |  |  |

Source: Aggregated base-year sector and product data from RIAPA’s Nexus SAMs.

Note: Export-intensity is the share of exports in total output; and import-intensity is share of imports in total demand. GDP is total gross domestic product.

Table S10: Malawi Production and Trade Structure

|  | Share of total value (%) | | | Export-intensity (%) | Import-intensity (%) |
| --- | --- | --- | --- | --- | --- |
|  | GDP | Exports | Imports |  |  |
|  |  |  |  |  |  |
| All sectors | 100.0 | 100.0 | 100.0 | 9.6 | 27.4 |
|  |  |  |  |  |  |
| Agriculture | 29.1 | 27.0 | 5.5 | 16.4 | 8.7 |
| Crops | 16.8 | 27.0 | 5.3 | 30.9 | 15.2 |
| *of which* Maize | 5.1 | 0.4 | 0.6 | 2.0 | 7.7 |
| Sorghum and millet | 0.4 | 0.0 | 0.0 | 1.5 | 0.0 |
| Rice | 0.5 | 0.0 | 0.0 | 0.0 | 0.3 |
| Wheat and barley | 0.0 | 0.0 | 1.5 | 0.0 | 99.5 |
| Other oilseeds | 0.4 | 2.1 | 0.3 | 88.5 | 70.3 |
| Livestock | 2.8 | 0.1 | 0.2 | 0.3 | 2.4 |
| Other agriculture | 9.4 | 0.0 | 0.0 | 0.0 | 0.2 |
|  |  |  |  |  |  |
| Mining | 1.5 | 5.0 | 1.1 | 55.0 | 38.0 |
| *of which* Crude oil | 0.0 | 0.0 | 0.0 | 0.0 | 0.0 |
| Natural gas | 0.0 | 0.0 | 0.0 | 0.0 | 0.0 |
|  |  |  |  |  |  |
| Manufacturing | 9.4 | 60.7 | 82.1 | 31.5 | 69.5 |
| *of which* Agro-processing | 6.6 | 53.5 | 6.2 | 32.5 | 18.5 |
| *of which* Maize milling | 0.0 | 0.0 | 0.0 | 0.0 | 0.0 |
| Sorghum & millet milling | 0.0 | 0.0 | 0.0 | 0.0 | 0.0 |
| Rice milling | 0.0 | 0.0 | 0.0 | 0.0 | 0.0 |
| Wheat & barley milling | 0.0 | 0.0 | 0.0 | 0.0 | 0.0 |
| Fats and oils | 0.0 | 0.1 | 0.5 | 5.8 | 48.4 |
| Other manufacturing | 2.8 | 7.2 | 75.9 | 27.4 | 89.7 |
| Petroleum | 0.0 | 0.0 | 14.7 | 0.0 | 100.0 |
| Fertilizers | 0.0 | 0.0 | 4.2 | 0.0 | 100.0 |
|  |  |  |  |  |  |
| Other industry | 5.6 | 0.0 | 0.0 | 0.0 | 0.0 |
|  |  |  |  |  |  |
| Services | 54.4 | 7.2 | 11.2 | 1.8 | 5.9 |
| Trade and hotels | 19.0 | 2.8 | 2.4 | 2.1 | 3.9 |
| Transport and communication | 7.1 | 2.0 | 3.0 | 3.9 | 12.4 |
| Finance and business services | 15.0 | 1.8 | 4.2 | 1.9 | 8.7 |
| Government services | 8.5 | 0.5 | 1.1 | 0.6 | 2.5 |
| Other services | 4.9 | 0.1 | 0.5 | 0.3 | 3.8 |
|  |  |  |  |  |  |

Source: Aggregated base-year sector and product data from RIAPA’s Nexus SAMs.

Note: Export-intensity is the share of exports in total output; and import-intensity is share of imports in total demand. GDP is total gross domestic product.

Table S11: Niger Production and Trade Structure

|  | Share of total value (%) | | | Export-intensity (%) | Import-intensity (%) |
| --- | --- | --- | --- | --- | --- |
|  | GDP | Exports | Imports |  |  |
|  |  |  |  |  |  |
| All sectors | 100.0 | 100.0 | 100.0 | 3.8 | 15.6 |
|  |  |  |  |  |  |
| Agriculture | 39.3 | 16.1 | 0.8 | 2.9 | 0.7 |
| Crops | 29.2 | 12.2 | 0.8 | 3.7 | 1.0 |
| *of which* Maize | 0.1 | 0.0 | 0.1 | 0.0 | 32.6 |
| Sorghum and millet | 14.1 | 0.0 | 0.1 | 0.0 | 0.6 |
| Rice | 0.4 | 0.0 | 0.0 | 0.0 | 2.5 |
| Wheat and barley | 0.0 | 0.0 | 0.3 | 0.0 | 71.9 |
| Other oilseeds | 0.2 | 0.0 | 0.0 | 0.7 | 0.2 |
| Livestock | 6.3 | 3.9 | 0.0 | 2.4 | 0.0 |
| Other agriculture | 3.8 | 0.0 | 0.0 | 0.0 | 0.0 |
|  |  |  |  |  |  |
| Mining | 7.6 | 38.5 | 0.9 | 22.3 | 2.4 |
| *of which* Crude oil | 3.2 | 0.0 | 0.0 | 0.0 | 0.0 |
| Natural gas | 0.0 | 0.0 | 0.0 | 0.0 | 0.0 |
|  |  |  |  |  |  |
| Manufacturing | 7.7 | 26.0 | 65.6 | 4.9 | 40.1 |
| *of which* Agro-processing | 2.7 | 2.7 | 16.5 | 2.0 | 39.5 |
| *of which* Maize milling | 0.0 | 0.0 | 0.1 | 0.0 | 94.3 |
| Sorghum & millet milling | 0.0 | 0.0 | 0.0 | 0.0 | 0.0 |
| Rice milling | 0.0 | 0.0 | 6.3 | 0.0 | 99.1 |
| Wheat & barley milling | 0.0 | 0.0 | 0.7 | 0.0 | 99.3 |
| Fats and oils | 0.4 | 0.1 | 0.5 | 0.4 | 7.9 |
| Other manufacturing | 5.0 | 23.3 | 49.1 | 5.8 | 40.4 |
| Petroleum | 1.5 | 20.6 | 2.0 | 14.7 | 8.4 |
| Fertilizers | 0.0 | 0.0 | 0.4 | 0.0 | 100.0 |
|  |  |  |  |  |  |
| Other industry | 6.4 | 0.0 | 4.1 | 0.0 | 5.2 |
|  |  |  |  |  |  |
| Services | 39.0 | 19.5 | 28.6 | 1.9 | 9.8 |
| Trade and hotels | 14.3 | 0.0 | 3.7 | 0.0 | 3.8 |
| Transport and communication | 4.9 | 1.8 | 22.4 | 0.8 | 28.2 |
| Finance and business services | 6.0 | 14.9 | 2.3 | 9.9 | 6.0 |
| Government services | 12.1 | 2.7 | 0.2 | 1.1 | 0.3 |
| Other services | 1.7 | 0.0 | 0.0 | 0.0 | 0.0 |
|  |  |  |  |  |  |

Source: Aggregated base-year sector and product data from RIAPA’s Nexus SAMs.

Note: Export-intensity is the share of exports in total output; and import-intensity is share of imports in total demand. GDP is total gross domestic product.

Table S12: Nigeria Production and Trade Structure

|  | Share of total value (%) | | | Export-intensity (%) | Import-intensity (%) |
| --- | --- | --- | --- | --- | --- |
|  | GDP | Exports | Imports |  |  |
|  |  |  |  |  |  |
| All sectors | 100.0 | 100.0 | 100.0 | 6.7 | 11.0 |
|  |  |  |  |  |  |
| Agriculture | 22.1 | 1.8 | 1.6 | 1.0 | 1.5 |
| Crops | 19.8 | 1.8 | 1.4 | 1.1 | 1.5 |
| *of which* Maize | 0.7 | 0.0 | 0.1 | 0.0 | 1.7 |
| Sorghum and millet | 0.7 | 0.0 | 0.0 | 0.0 | 0.0 |
| Rice | 1.6 | 0.0 | 0.0 | 0.0 | 0.0 |
| Wheat and barley | 0.0 | 0.0 | 1.0 | 0.0 | 96.1 |
| Other oilseeds | 0.6 | 0.5 | 0.0 | 5.0 | 0.0 |
| Livestock | 1.4 | 0.0 | 0.1 | 0.0 | 0.6 |
| Other agriculture | 0.8 | 0.0 | 0.1 | 0.0 | 2.0 |
|  |  |  |  |  |  |
| Mining | 8.9 | 87.5 | 0.2 | 82.5 | 1.9 |
| *of which* Crude oil | 7.6 | 76.5 | 0.0 | 84.1 | 0.0 |
| Natural gas | 1.1 | 10.9 | 0.0 | 100.0 | 0.0 |
|  |  |  |  |  |  |
| Manufacturing | 11.6 | 5.4 | 60.4 | 1.8 | 27.6 |
| *of which* Agro-processing | 4.3 | 0.3 | 3.8 | 0.4 | 9.2 |
| *of which* Maize milling | 0.1 | 0.0 | 0.0 | 0.0 | 1.2 |
| Sorghum & millet milling | 0.0 | 0.0 | 0.0 | 0.0 | 0.0 |
| Rice milling | 0.3 | 0.0 | 0.0 | 0.0 | 0.0 |
| Wheat & barley milling | 0.0 | 0.0 | 0.0 | 0.0 | 1.6 |
| Fats and oils | 0.0 | 0.0 | 0.4 | 3.1 | 57.8 |
| Other manufacturing | 7.4 | 5.2 | 56.5 | 2.3 | 32.4 |
| Petroleum | 0.1 | 1.0 | 11.3 | 9.8 | 66.9 |
| Fertilizers | 0.2 | 0.3 | 0.4 | 5.9 | 15.5 |
|  |  |  |  |  |  |
| Other industry | 7.2 | 0.2 | 0.0 | 0.1 | 0.0 |
|  |  |  |  |  |  |
| Services | 50.3 | 5.1 | 37.9 | 0.7 | 7.3 |
| Trade and hotels | 16.6 | 0.2 | 11.7 | 0.1 | 8.6 |
| Transport and communication | 12.8 | 3.0 | 6.7 | 1.0 | 3.2 |
| Finance and business services | 12.7 | 1.2 | 19.3 | 0.9 | 17.1 |
| Government services | 4.7 | 0.7 | 0.3 | 1.2 | 0.7 |
| Other services | 3.5 | 0.0 | 0.0 | 0.0 | 0.0 |
|  |  |  |  |  |  |

Source: Aggregated base-year sector and product data from RIAPA’s Nexus SAMs.

Note: Export-intensity is the share of exports in total output; and import-intensity is share of imports in total demand. GDP is total gross domestic product.

Table S13: Nepal Production and Trade Structure

|  | Share of total value (%) | | | Export-intensity (%) | Import-intensity (%) |
| --- | --- | --- | --- | --- | --- |
|  | GDP | Exports | Imports |  |  |
|  |  |  |  |  |  |
| All sectors | 100.0 | 100.0 | 100.0 | 3.2 | 17.2 |
|  |  |  |  |  |  |
| Agriculture | 30.0 | 5.2 | 6.6 | 0.5 | 4.4 |
| Crops | 21.8 | 5.1 | 6.3 | 0.7 | 5.5 |
| *of which* Maize | 1.4 | 0.0 | 0.6 | 0.0 | 8.0 |
| Sorghum and millet | 0.3 | 0.0 | 0.0 | 0.0 | 1.6 |
| Rice | 7.6 | 0.0 | 0.5 | 0.0 | 1.6 |
| Wheat and barley | 1.7 | 0.0 | 0.4 | 0.0 | 4.7 |
| Other oilseeds | 1.4 | 0.0 | 0.8 | 0.0 | 10.8 |
| Livestock | 7.4 | 0.1 | 0.2 | 0.0 | 0.7 |
| Other agriculture | 0.8 | 0.0 | 0.1 | 0.0 | 1.9 |
|  |  |  |  |  |  |
| Mining | 0.6 | 0.1 | 2.6 | 0.6 | 49.5 |
| *of which* Crude oil | 0.0 | 0.0 | 0.0 | 0.0 | 1.1 |
| Natural gas | 0.0 | 0.0 | 0.0 | 0.0 | 0.0 |
|  |  |  |  |  |  |
| Manufacturing | 5.8 | 20.9 | 77.1 | 4.3 | 51.9 |
| *of which* Agro-processing | 2.5 | 4.1 | 5.8 | 2.0 | 18.3 |
| *of which* Maize milling | 0.2 | 0.0 | 0.0 | 0.0 | 0.2 |
| Sorghum & millet milling | 0.0 | 0.0 | 0.0 | 0.0 | 0.0 |
| Rice milling | 0.4 | 0.0 | 1.7 | 0.0 | 25.9 |
| Wheat & barley milling | 0.0 | 0.0 | 0.0 | 1.2 | 0.4 |
| Fats and oils | 0.1 | 0.2 | 0.2 | 0.9 | 8.1 |
| Other manufacturing | 3.2 | 16.8 | 71.2 | 5.7 | 62.1 |
| Petroleum | 0.0 | 0.0 | 11.5 | 0.0 | 96.0 |
| Fertilizers | 0.0 | 0.0 | 1.6 | 0.0 | 88.9 |
|  |  |  |  |  |  |
| Other industry | 9.0 | 0.0 | 0.0 | 0.0 | 0.0 |
|  |  |  |  |  |  |
| Services | 54.6 | 73.7 | 13.7 | 5.4 | 5.5 |
| Trade and hotels | 16.5 | 27.2 | 6.4 | 6.5 | 8.2 |
| Transport and communication | 7.7 | 12.5 | 4.6 | 5.2 | 10.0 |
| Finance and business services | 13.4 | 16.0 | 2.5 | 5.4 | 4.6 |
| Government services | 12.0 | 18.0 | 0.2 | 6.1 | 0.4 |
| Other services | 4.9 | 0.0 | 0.0 | 0.0 | 0.0 |
|  |  |  |  |  |  |

Source: Aggregated base-year sector and product data from RIAPA’s Nexus SAMs.

Note: Export-intensity is the share of exports in total output; and import-intensity is share of imports in total demand. GDP is total gross domestic product.

Table S14: Philippines Production and Trade Structure

|  | Share of total value (%) | | | Export-intensity (%) | Import-intensity (%) |
| --- | --- | --- | --- | --- | --- |
|  | GDP | Exports | Imports |  |  |
|  |  |  |  |  |  |
| All sectors | 100.0 | 100.0 | 100.0 | 11.3 | 18.1 |
|  |  |  |  |  |  |
| Agriculture | 8.4 | 2.9 | 1.9 | 3.9 | 5.6 |
| Crops | 5.2 | 2.7 | 1.9 | 7.2 | 10.3 |
| *of which* Maize | 0.3 | 0.0 | 0.1 | 0.0 | 3.3 |
| Sorghum and millet | 0.0 | 0.0 | 0.0 | 0.0 | 0.0 |
| Rice | 1.7 | 0.0 | 0.0 | 0.0 | 0.0 |
| Wheat and barley | 0.0 | 0.0 | 1.6 | 0.0 | 98.3 |
| Other oilseeds | 0.4 | 0.2 | 0.1 | 11.7 | 7.6 |
| Livestock | 1.9 | 0.0 | 0.0 | 0.0 | 0.1 |
| Other agriculture | 1.2 | 0.1 | 0.0 | 1.0 | 0.2 |
|  |  |  |  |  |  |
| Mining | 0.8 | 3.0 | 4.8 | 34.3 | 61.2 |
| *of which* Crude oil | 0.1 | 0.2 | 3.3 | 39.6 | 96.1 |
| Natural gas | 0.2 | 0.0 | 0.0 | 0.0 | 0.0 |
|  |  |  |  |  |  |
| Manufacturing | 18.3 | 62.6 | 75.2 | 25.3 | 40.1 |
| *of which* Agro-processing | 8.8 | 3.6 | 6.0 | 3.2 | 10.5 |
| *of which* Maize milling | 0.1 | 0.0 | 0.0 | 0.0 | 0.0 |
| Sorghum & millet milling | 0.0 | 0.0 | 0.0 | 0.0 | 0.0 |
| Rice milling | 1.4 | 0.0 | 1.9 | 0.0 | 20.4 |
| Wheat & barley milling | 0.5 | 0.0 | 0.0 | 0.0 | 0.0 |
| Fats and oils | 1.9 | 0.0 | 0.0 | 0.0 | 0.7 |
| Other manufacturing | 9.5 | 58.9 | 69.2 | 43.6 | 59.4 |
| Petroleum | 1.0 | 0.3 | 7.6 | 2.0 | 49.0 |
| Fertilizers | 0.1 | 0.0 | 1.2 | 2.7 | 68.3 |
|  |  |  |  |  |  |
| Other industry | 11.3 | 0.0 | 0.0 | 0.0 | 0.0 |
|  |  |  |  |  |  |
| Services | 61.3 | 31.6 | 18.1 | 6.9 | 6.2 |
| Trade and hotels | 20.1 | 0.0 | 4.9 | 0.0 | 5.1 |
| Transport and communication | 6.5 | 4.1 | 4.2 | 6.4 | 9.9 |
| Finance and business services | 21.7 | 27.5 | 8.6 | 16.9 | 9.1 |
| Government services | 10.2 | 0.0 | 0.4 | 0.0 | 1.1 |
| Other services | 2.9 | 0.0 | 0.0 | 0.0 | 0.0 |
|  |  |  |  |  |  |

Source: Aggregated base-year sector and product data from RIAPA’s Nexus SAMs.

Note: Export-intensity is the share of exports in total output; and import-intensity is share of imports in total demand. GDP is total gross domestic product.

Table S15: Rwanda Production and Trade Structure

|  | Share of total value (%) | | | Export-intensity (%) | Import-intensity (%) |
| --- | --- | --- | --- | --- | --- |
|  | GDP | Exports | Imports |  |  |
|  |  |  |  |  |  |
| All sectors | 100.0 | 100.0 | 100.0 | 13.1 | 24.4 |
|  |  |  |  |  |  |
| Agriculture | 25.7 | 15.5 | 0.8 | 17.0 | 2.1 |
| Crops | 16.0 | 15.5 | 0.8 | 22.6 | 3.0 |
| *of which* Maize | 0.7 | 0.0 | 0.3 | 0.0 | 18.7 |
| Sorghum and millet | 0.5 | 0.0 | 0.0 | 0.0 | 0.0 |
| Rice | 0.5 | 0.0 | 0.0 | 0.0 | 0.0 |
| Wheat and barley | 0.0 | 0.0 | 0.3 | 0.0 | 85.1 |
| Other oilseeds | 0.1 | 0.0 | 0.0 | 0.0 | 13.2 |
| Livestock | 2.9 | 0.0 | 0.0 | 0.0 | 0.2 |
| Other agriculture | 6.8 | 0.0 | 0.0 | 0.0 | 0.0 |
|  |  |  |  |  |  |
| Mining | 1.6 | 12.7 | 0.0 | 70.9 | 0.1 |
| *of which* Crude oil | 0.0 | 0.0 | 0.0 | 0.0 | 100.0 |
| Natural gas | 0.1 | 0.0 | 0.0 | 0.0 | 0.0 |
|  |  |  |  |  |  |
| Manufacturing | 9.2 | 17.6 | 73.6 | 17.2 | 63.9 |
| *of which* Agro-processing | 5.3 | 10.7 | 5.7 | 18.6 | 18.9 |
| *of which* Maize milling | 0.0 | 0.0 | 0.0 | 0.0 | 0.0 |
| Sorghum & millet milling | 0.0 | 0.0 | 0.0 | 0.0 | 0.0 |
| Rice milling | 0.4 | 0.0 | 0.7 | 0.0 | 16.4 |
| Wheat & barley milling | 0.0 | 0.0 | 0.0 | 0.0 | 0.0 |
| Fats and oils | 0.2 | 0.0 | 0.2 | 0.0 | 39.6 |
| Other manufacturing | 3.9 | 6.9 | 67.9 | 15.2 | 78.0 |
| Petroleum | 0.0 | 0.0 | 17.3 | 0.0 | 99.8 |
| Fertilizers | 0.0 | 0.0 | 3.2 | 0.0 | 100.0 |
|  |  |  |  |  |  |
| Other industry | 9.9 | 0.0 | 0.0 | 0.0 | 0.0 |
|  |  |  |  |  |  |
| Services | 53.6 | 54.2 | 25.5 | 13.6 | 11.3 |
| Trade and hotels | 11.9 | 3.6 | 2.4 | 3.4 | 3.8 |
| Transport and communication | 8.8 | 34.1 | 21.0 | 46.9 | 48.2 |
| Finance and business services | 16.3 | 1.8 | 1.3 | 2.2 | 2.7 |
| Government services | 11.0 | 14.8 | 0.0 | 14.0 | 0.0 |
| Other services | 5.6 | 0.0 | 0.8 | 0.0 | 4.1 |
|  |  |  |  |  |  |

Source: Aggregated base-year sector and product data from RIAPA’s Nexus SAMs.

Note: Export-intensity is the share of exports in total output; and import-intensity is share of imports in total demand. GDP is total gross domestic product.

Table S16: Senegal Production and Trade Structure

|  | Share of total value (%) | | | Export-intensity (%) | Import-intensity (%) |
| --- | --- | --- | --- | --- | --- |
|  | GDP | Exports | Imports |  |  |
|  |  |  |  |  |  |
| All sectors | 100.0 | 100.0 | 100.0 | 9.2 | 18.7 |
|  |  |  |  |  |  |
| Agriculture | 16.5 | 13.2 | 5.4 | 10.3 | 9.1 |
| Crops | 9.6 | 11.8 | 5.2 | 15.0 | 14.3 |
| *of which* Maize | 0.4 | 0.0 | 1.1 | 0.0 | 43.2 |
| Sorghum and millet | 1.4 | 0.0 | 0.0 | 0.0 | 0.0 |
| Rice | 1.9 | 0.0 | 0.0 | 0.0 | 0.0 |
| Wheat and barley | 0.0 | 0.0 | 2.6 | 0.0 | 100.0 |
| Other oilseeds | 1.0 | 0.0 | 0.1 | 0.0 | 1.7 |
| Livestock | 4.2 | 0.0 | 0.2 | 0.2 | 1.3 |
| Other agriculture | 2.6 | 1.4 | 0.0 | 6.6 | 0.0 |
|  |  |  |  |  |  |
| Mining | 4.2 | 10.3 | 5.7 | 32.4 | 32.9 |
| *of which* Crude oil | 0.0 | 0.0 | 4.9 | 0.0 | 100.0 |
| Natural gas | 0.0 | 0.0 | 0.0 | 0.0 | 0.0 |
|  |  |  |  |  |  |
| Manufacturing | 16.6 | 43.0 | 68.7 | 12.6 | 35.4 |
| *of which* Agro-processing | 8.8 | 20.1 | 12.3 | 13.6 | 19.0 |
| *of which* Maize milling | 0.2 | 0.0 | 0.0 | 0.0 | 1.5 |
| Sorghum & millet milling | 0.4 | 0.0 | 0.0 | 0.0 | 0.0 |
| Rice milling | 0.4 | 0.0 | 2.6 | 0.0 | 46.5 |
| Wheat & barley milling | 0.0 | 0.0 | 0.1 | 0.0 | 100.0 |
| Fats and oils | 0.5 | 0.1 | 0.7 | 1.4 | 15.4 |
| Other manufacturing | 7.8 | 22.9 | 56.4 | 11.9 | 44.4 |
| Petroleum | 0.4 | 0.0 | 6.9 | 0.0 | 48.7 |
| Fertilizers | 0.1 | 0.0 | 0.7 | 0.0 | 57.0 |
|  |  |  |  |  |  |
| Other industry | 5.5 | 2.4 | 0.7 | 2.2 | 1.1 |
|  |  |  |  |  |  |
| Services | 57.2 | 31.1 | 19.6 | 7.0 | 7.9 |
| Trade and hotels | 16.3 | 11.2 | 2.1 | 8.7 | 3.2 |
| Transport and communication | 8.6 | 4.4 | 10.0 | 4.9 | 17.6 |
| Finance and business services | 17.3 | 10.3 | 6.6 | 8.6 | 9.7 |
| Government services | 12.7 | 5.0 | 0.7 | 5.3 | 1.5 |
| Other services | 2.2 | 0.2 | 0.1 | 1.3 | 1.9 |
|  |  |  |  |  |  |

Source: Aggregated base-year sector and product data from RIAPA’s Nexus SAMs.

Note: Export-intensity is the share of exports in total output; and import-intensity is share of imports in total demand. GDP is total gross domestic product.

Table S17: Tanzania Production and Trade Structure

|  | Share of total value (%) | | | Export-intensity (%) | Import-intensity (%) |
| --- | --- | --- | --- | --- | --- |
|  | GDP | Exports | Imports |  |  |
|  |  |  |  |  |  |
| All sectors | 100.0 | 100.0 | 100.0 | 9.0 | 11.8 |
|  |  |  |  |  |  |
| Agriculture | 28.9 | 7.9 | 0.5 | 3.7 | 0.4 |
| Crops | 16.1 | 7.6 | 0.5 | 6.4 | 0.6 |
| *of which* Maize | 4.1 | 0.6 | 0.3 | 2.5 | 1.5 |
| Sorghum and millet | 0.3 | 0.2 | 0.0 | 11.4 | 0.7 |
| Rice | 1.9 | 0.0 | 0.0 | 0.1 | 0.0 |
| Wheat and barley | 0.1 | 0.2 | 0.0 | 21.5 | 11.1 |
| Other oilseeds | 1.6 | 1.6 | 0.0 | 13.5 | 0.0 |
| Livestock | 7.4 | 0.2 | 0.0 | 0.4 | 0.1 |
| Other agriculture | 5.4 | 0.1 | 0.0 | 0.1 | 0.0 |
|  |  |  |  |  |  |
| Mining | 5.6 | 25.4 | 0.2 | 45.2 | 0.9 |
| *of which* Crude oil | 0.4 | 0.0 | 0.0 | 0.0 | 0.0 |
| Natural gas | 0.0 | 0.0 | 0.0 | 0.0 | 0.0 |
|  |  |  |  |  |  |
| Manufacturing | 9.2 | 20.8 | 82.6 | 10.1 | 38.2 |
| *of which* Agro-processing | 2.9 | 10.7 | 3.6 | 12.9 | 7.1 |
| *of which* Maize milling | 0.2 | 0.2 | 0.0 | 2.1 | 0.0 |
| Sorghum & millet milling | 0.0 | 0.1 | 0.0 | 10.7 | 0.0 |
| Rice milling | 0.3 | 0.5 | 0.0 | 3.1 | 0.0 |
| Wheat & barley milling | 0.0 | 0.2 | 0.1 | 26.3 | 19.6 |
| Fats and oils | 0.0 | 0.5 | 0.1 | 92.3 | 72.5 |
| Other manufacturing | 6.3 | 10.1 | 79.0 | 8.3 | 48.7 |
| Petroleum | 0.0 | 1.5 | 17.9 | 91.3 | 90.9 |
| Fertilizers | 0.0 | 0.0 | 2.3 | 0.0 | 85.7 |
|  |  |  |  |  |  |
| Other industry | 16.3 | 0.3 | 0.0 | 0.1 | 0.0 |
|  |  |  |  |  |  |
| Services | 40.0 | 45.6 | 16.6 | 11.5 | 4.9 |
| Trade and hotels | 10.9 | 11.1 | 3.0 | 9.9 | 3.1 |
| Transport and communication | 9.1 | 19.6 | 8.0 | 18.2 | 9.1 |
| Finance and business services | 10.4 | 13.2 | 4.7 | 13.4 | 5.7 |
| Government services | 8.2 | 1.5 | 1.0 | 2.3 | 1.7 |
| Other services | 1.4 | 0.2 | 0.0 | 1.6 | 0.0 |
|  |  |  |  |  |  |

Source: Aggregated base-year sector and product data from RIAPA’s Nexus SAMs.

Note: Export-intensity is the share of exports in total output; and import-intensity is share of imports in total demand. GDP is total gross domestic product.

Table S18: Uganda Production and Trade Structure

|  | Share of total value (%) | | | Export-intensity (%) | Import-intensity (%) |
| --- | --- | --- | --- | --- | --- |
|  | GDP | Exports | Imports |  |  |
|  |  |  |  |  |  |
| All sectors | 100.0 | 100.0 | 100.0 | 9.2 | 15.5 |
|  |  |  |  |  |  |
| Agriculture | 24.8 | 19.2 | 1.5 | 11.6 | 1.7 |
| Crops | 14.8 | 19.1 | 1.4 | 21.3 | 3.3 |
| *of which* Maize | 1.2 | 2.0 | 0.0 | 22.2 | 0.0 |
| Sorghum and millet | 0.4 | 0.9 | 0.0 | 42.9 | 0.0 |
| Rice | 0.2 | 0.0 | 0.1 | 0.4 | 8.3 |
| Wheat and barley | 0.0 | 0.0 | 0.8 | 0.0 | 92.5 |
| Other oilseeds | 0.9 | 1.7 | 0.0 | 41.5 | 3.7 |
| Livestock | 3.1 | 0.1 | 0.1 | 0.2 | 0.4 |
| Other agriculture | 6.8 | 0.0 | 0.0 | 0.0 | 0.0 |
|  |  |  |  |  |  |
| Mining | 1.9 | 0.2 | 0.5 | 0.6 | 3.4 |
| *of which* Crude oil | 0.0 | 0.0 | 0.0 | 0.0 | 5.1 |
| Natural gas | 0.0 | 0.0 | 0.0 | 0.0 | 0.0 |
|  |  |  |  |  |  |
| Manufacturing | 16.7 | 36.9 | 58.5 | 15.1 | 35.4 |
| *of which* Agro-processing | 8.4 | 23.5 | 6.7 | 19.6 | 11.0 |
| *of which* Maize milling | 0.7 | 0.9 | 0.0 | 8.6 | 0.0 |
| Sorghum & millet milling | 0.2 | 0.4 | 0.0 | 15.4 | 0.0 |
| Rice milling | 0.2 | 0.9 | 1.0 | 30.7 | 41.0 |
| Wheat & barley milling | 0.1 | 0.7 | 0.1 | 50.5 | 11.2 |
| Fats and oils | 0.1 | 1.8 | 0.0 | 75.1 | 12.6 |
| Other manufacturing | 8.3 | 13.4 | 51.8 | 10.4 | 47.7 |
| Petroleum | 0.0 | 0.0 | 11.5 | 0.0 | 90.3 |
| Fertilizers | 0.1 | 0.0 | 0.9 | 0.0 | 52.2 |
|  |  |  |  |  |  |
| Other industry | 10.2 | 3.5 | 1.5 | 2.6 | 1.5 |
|  |  |  |  |  |  |
| Services | 46.5 | 40.2 | 38.0 | 8.5 | 10.9 |
| Trade and hotels | 12.5 | 12.0 | 2.7 | 9.5 | 3.2 |
| Transport and communication | 5.7 | 3.2 | 21.9 | 5.5 | 35.3 |
| Finance and business services | 13.8 | 5.0 | 9.9 | 4.0 | 10.3 |
| Government services | 11.0 | 0.0 | 0.0 | 0.0 | 0.0 |
| Other services | 3.6 | 20.0 | 3.5 | 47.5 | 18.2 |
|  |  |  |  |  |  |

Source: Aggregated base-year sector and product data from RIAPA’s Nexus SAMs.

Note: Export-intensity is the share of exports in total output; and import-intensity is share of imports in total demand. GDP is total gross domestic product.

Table S19: Zambia Production and Trade Structure

|  | Share of total value (%) | | | Export-intensity (%) | Import-intensity (%) |
| --- | --- | --- | --- | --- | --- |
|  | GDP | Exports | Imports |  |  |
|  |  |  |  |  |  |
| All sectors | 100.0 | 100.0 | 100.0 | 19.9 | 19.2 |
|  |  |  |  |  |  |
| Agriculture | 3.1 | 2.5 | 1.0 | 12.3 | 6.6 |
| Crops | 1.4 | 2.2 | 0.7 | 30.3 | 14.2 |
| *of which* Maize | 0.4 | 0.6 | 0.1 | 35.6 | 5.0 |
| Sorghum and millet | 0.0 | 0.0 | 0.0 | 0.0 | 11.8 |
| Rice | 0.0 | 0.0 | 0.1 | 0.8 | 28.8 |
| Wheat and barley | 0.1 | 0.0 | 0.1 | 0.3 | 26.8 |
| Other oilseeds | 0.1 | 0.1 | 0.0 | 51.7 | 4.7 |
| Livestock | 0.7 | 0.3 | 0.1 | 4.5 | 1.4 |
| Other agriculture | 0.9 | 0.1 | 0.2 | 1.4 | 2.9 |
|  |  |  |  |  |  |
| Mining | 15.8 | 2.3 | 10.8 | 11.9 | 33.8 |
| *of which* Crude oil | 0.0 | 0.0 | 5.7 | 0.0 | 100.0 |
| Natural gas | 0.0 | 0.0 | 0.0 | 0.0 | 0.0 |
|  |  |  |  |  |  |
| Manufacturing | 8.0 | 87.3 | 66.1 | 64.1 | 51.9 |
| *of which* Agro-processing | 2.6 | 4.9 | 4.6 | 16.3 | 15.7 |
| *of which* Maize milling | 0.1 | 0.0 | 0.0 | 0.0 | 0.0 |
| Sorghum & millet milling | 0.0 | 0.0 | 0.0 | 0.0 | 0.0 |
| Rice milling | 0.1 | 0.0 | 0.2 | 0.0 | 18.0 |
| Wheat & barley milling | 0.1 | 0.0 | 0.0 | 0.0 | 0.0 |
| Fats and oils | 0.0 | 0.1 | 0.2 | 37.9 | 35.2 |
| Other manufacturing | 5.4 | 82.4 | 61.4 | 73.1 | 63.3 |
| Petroleum | 0.4 | 0.0 | 11.3 | 0.0 | 65.8 |
| Fertilizers | 0.0 | 0.0 | 0.2 | 0.0 | 100.0 |
|  |  |  |  |  |  |
| Other industry | 17.8 | 1.1 | 0.0 | 0.7 | 0.0 |
|  |  |  |  |  |  |
| Services | 55.3 | 6.8 | 22.1 | 3.1 | 6.4 |
| Trade and hotels | 18.5 | 1.5 | 8.0 | 2.6 | 8.6 |
| Transport and communication | 12.9 | 2.8 | 8.0 | 5.4 | 9.8 |
| Finance and business services | 12.9 | 2.3 | 5.2 | 4.4 | 6.1 |
| Government services | 10.3 | 0.0 | 0.9 | 0.0 | 1.2 |
| Other services | 0.8 | 0.2 | 0.0 | 3.4 | 0.0 |
|  |  |  |  |  |  |

Source: Aggregated base-year sector and product data from RIAPA’s Nexus SAMs.

Note: Export-intensity is the share of exports in total output; and import-intensity is share of imports in total demand. GDP is total gross domestic product.

Table S20. Trade Substitution and Transformation Function Elasticities

| Product category | Elasticity | | Product category | Elasticity | |
| --- | --- | --- | --- | --- | --- |
|  | |  |  | |  |
| Rice | | 5.05 | Fats and oils | | 3.30 |
| Wheat and barley | | 4.45 | Dairy | | 3.30 |
| Other cereals | | 1.30 | Milled grains (incl. wheat flour) | | 2.60 |
| Pulses | | 1.85 | Refined sugar | | 2.70 |
| Groundnuts and oilseeds | | 2.45 | Coffee and tea (processed) | | 2.00 |
| Potatoes and root crops | | 1.85 | Other foods (incl. animal feed) | | 2.00 |
| Vegetables | | 3.25 | Beverages and tobacco | | 1.15 |
| Sugarcane | | 2.70 | Textiles and yarn | | 3.75 |
| Tobacco | | 3.25 | Clothing | | 3.70 |
| Cotton and fibers | | 2.50 | Leather and footwear | | 4.05 |
| Nuts and fruits | | 1.85 | Wood products | | 3.40 |
| Beverage crops | | 3.25 | Paper products and printing | | 2.95 |
| Rubber | | 2.50 | Petroleum products | | 2.10 |
| Other crops | | 3.25 | Fertilizers and herbicides | | 3.30 |
| Cattle | | 2.00 | Other chemical products | | 3.30 |
| Raw milk | | 3.65 | Nonmetallic minerals | | 2.90 |
| Poultry and eggs | | 1.30 | Basic metals | | 2.95 |
| Small ruminants | | 2.00 | Metal products | | 3.75 |
| Other livestock | | 1.30 | Electrical equipment | | 4.05 |
| Forestry | | 2.50 | Machinery and other equipment | | 4.40 |
| Fisheries | | 1.25 | Motor vehicles | | 4.30 |
| Mining products (incl. crude oil) | | 0.50 | Other manufacturing | | 3.75 |
| Meats (processed) | | 3.85 | Electricity, gas and steam | | 2.80 |
| Fish and seafood (processed) | | 4.40 | Water supply and sewage | | 2.80 |
| Fruit and vegetables (processed) | | 3.65 | Construction and services | | 0.50 |
|  | |  |  | |  |

Source: Authors adaptation of GTAP elasticities from Dimaranan (2006).

Note: Export-intensity is the share of exports in total output; and import-intensity is share of imports in total demand. GDP is total gross domestic product.

Table S21. Country Fertilizer Use by Crop (FUBC) Estimates

|  | Share of cultivated crop land grown using inorganic/chemical fertilizers (%) | | | | | | | | | |
| --- | --- | --- | --- | --- | --- | --- | --- | --- | --- | --- |
|  | Bangladesh | Cambodia | DRC | Egypt | Ethiopia | Ghana | Kenya | Mali | Myanmar | Malawi |
|  |  |  |  |  |  |  |  |  |  |  |
| Maize | 85.0 | 74.3 | 70.0 | 100.0 | 88.5 | 70.5 | 71.2 | 75.3 | 75.7 | 71.3 |
| Sorghum and millet | 0.0 | 0.0 | 2.0 | 100.0 | 33.9 | 32.3 | 35.5 | 34.0 | 52.4 | 28.1 |
| Rice | 88.0 | 83.1 | 60.0 | 100.0 | 89.4 | 60.7 | 93.8 | 44.0 | 80.6 | 23.0 |
| Wheat and barley | 90.0 | 0.0 | 60.0 | 100.0 | 92.6 | 0.0 | 91.4 | 0.0 | 58.6 | 91.4 |
| Pulses | 85.0 | 93.8 | 30.0 | 100.0 | 34.3 | 34.3 | 26.7 | 29.5 | 56.4 | 73.9 |
| Groundnuts | 0.0 | 59.0 | 8.0 | 100.0 | 24.8 | 39.2 | 0.0 | 6.7 | 65.3 | 10.8 |
| Other oilseeds | 90.0 | 65.8 | 15.0 | 100.0 | 17.2 | 48.5 | 0.0 | 9.9 | 61.7 | 14.2 |
| Cassava | 0.0 | 65.0 | 50.0 | 0.0 | 0.0 | 48.3 | 0.0 | 64.0 | 34.0 | 0.0 |
| Irish potatoes | 85.0 | 66.5 | 40.0 | 0.0 | 86.4 | 66.7 | 77.6 | 45.2 | 72.4 | 0.0 |
| Sweet potatoes | 0.0 | 57.7 | 15.0 | 0.0 | 58.5 | 66.7 | 0.0 | 64.8 | 63.2 | 0.0 |
| Other vegetables | 78.0 | 79.3 | 60.0 | 95.0 | 85.4 | 7.2 | 64.3 | 37.8 | 75.0 | 70.4 |
| Sugarcane | 78.0 | 99.1 | 40.0 | 100.0 | 45.1 | 33.3 | 10.1 | 0.0 | 76.4 | 43.3 |
| Tobacco | 45.0 | 99.8 | 0.0 | 0.0 | 70.0 | 0.0 | 70.0 | 0.0 | 41.6 | 96.0 |
| Cotton and fibers | 95.0 | 0.0 | 0.0 | 100.0 | 47.4 | 65.0 | 47.4 | 100.0 | 53.3 | 1.4 |
| Bananas | 65.0 | 0.0 | 20.0 | 0.0 | 38.9 | 40.0 | 0.0 | 0.0 | 59.7 | 0.0 |
| Other fruits | 52.0 | 45.5 | 20.0 | 95.0 | 41.6 | 45.1 | 17.7 | 48.8 | 55.1 | 25.1 |
| Leaf tea | 90.0 | 56.5 | 0.0 | 0.0 | 38.8 | 0.0 | 100.0 | 0.0 | 51.5 | 70.4 |
| Coffee | 0.0 | 56.5 | 30.0 | 0.0 | 36.5 | 0.0 | 55.0 | 0.0 | 58.4 | 0.0 |
| Cocoa | 0.0 | 0.0 | 0.0 | 0.0 | 0.0 | 71.6 | 47.4 | 0.0 | 61.7 | 25.1 |
| Cut flowers | 100.0 | 0.0 | 0.0 | 0.0 | 100.0 | 0.0 | 100.0 | 0.0 | 76.9 | 0.0 |
| Rubber | 0.0 | 51.7 | 0.0 | 0.0 | 0.0 | 70.6 | 0.0 | 0.0 | 61.7 | 0.0 |
| Other crops | 0.0 | 56.5 | 0.0 | 95.0 | 0.0 | 0.0 | 0.0 | 0.0 | 61.7 | 0.0 |
|  |  |  |  |  |  |  |  |  |  |  |

Source: Authors estimates using on information from various sources, including Africa Fertilizer (2022), Ludemann et al. (2022), and data extracted from national farm and household surveys.

Note: Not all countries grow all the crops shown in the table, or those crops are not disaggregated in the model databases (these appears as zeros in the table).

Table S21 (continued). Country Fertilizer Use by Crop (FUBC) Estimates

|  | Share of cultivated crop land grown using inorganic/chemical fertilizers (%) | | | | | | | | | |
| --- | --- | --- | --- | --- | --- | --- | --- | --- | --- | --- |
|  | Niger | Nigeria | Nepal | Philippines | Rwanda | Senegal | Tanzania | Uganda | Zambia | Niger |
|  |  |  |  |  |  |  |  |  |  |  |
| Maize | 18.3 | 50.2 | 44.6 | 82.4 | 54.5 | 40.9 | 12.6 | 8.3 | 73.3 | 18.3 |
| Sorghum and millet | 0.0 | 57.9 | 20.5 | 0.0 | 32.3 | 27.2 | 0.6 | 0.0 | 49.3 | 0.0 |
| Rice | 78.9 | 63.8 | 80.0 | 99.8 | 64.6 | 70.8 | 7.1 | 6.4 | 37.6 | 78.9 |
| Wheat and barley | 65.7 | 0.0 | 57.0 | 54.3 | 74.3 | 0.0 | 4.2 | 16.6 | 0.0 | 65.7 |
| Pulses | 10.2 | 55.4 | 3.6 | 0.0 | 44.6 | 1.3 | 9.3 | 3.7 | 26.1 | 10.2 |
| Groundnuts | 0.0 | 38.6 | 0.0 | 0.0 | 37.7 | 29.4 | 0.8 | 1.1 | 8.2 | 0.0 |
| Other oilseeds | 0.0 | 56.6 | 13.1 | 65.8 | 37.5 | 0.0 | 1.2 | 0.0 | 8.2 | 0.0 |
| Cassava | 46.5 | 15.8 | 0.0 | 65.0 | 43.2 | 9.8 | 0.0 | 0.6 | 0.4 | 46.5 |
| Irish potatoes | 100.0 | 62.5 | 34.3 | 66.5 | 71.8 | 96.8 | 69.4 | 11.5 | 27.4 | 100.0 |
| Sweet potatoes | 0.0 | 48.1 | 0.0 | 57.7 | 42.2 | 80.5 | 1.4 | 1.0 | 2.3 | 0.0 |
| Other vegetables | 67.9 | 42.9 | 7.3 | 79.3 | 77.1 | 52.4 | 64.6 | 35.6 | 11.6 | 67.9 |
| Sugarcane | 0.0 | 48.5 | 73.8 | 80.0 | 48.0 | 0.0 | 22.5 | 2.1 | 84.8 | 0.0 |
| Tobacco | 0.0 | 0.0 | 0.0 | 95.0 | 70.2 | 0.0 | 35.9 | 57.6 | 84.8 | 0.0 |
| Cotton and fibers | 0.0 | 56.1 | 55.2 | 0.0 | 0.0 | 93.7 | 0.0 | 0.6 | 77.4 | 0.0 |
| Bananas | 0.0 | 0.0 | 6.7 | 45.5 | 41.2 | 0.0 | 0.9 | 1.1 | 0.0 | 0.0 |
| Other fruits | 91.6 | 18.3 | 0.0 | 45.5 | 54.5 | 42.7 | 4.6 | 9.0 | 37.0 | 91.6 |
| Leaf tea | 0.0 | 0.0 | 85.9 | 0.0 | 100.0 | 0.0 | 34.6 | 71.7 | 0.0 | 0.0 |
| Coffee | 0.0 | 0.0 | 0.0 | 56.5 | 48.7 | 0.0 | 17.2 | 2.6 | 37.0 | 0.0 |
| Cocoa | 0.0 | 25.0 | 0.0 | 0.0 | 0.0 | 0.0 | 0.0 | 0.0 | 0.0 | 0.0 |
| Cut flowers | 0.0 | 0.0 | 0.0 | 0.0 | 0.0 | 0.0 | 100.0 | 0.0 | 0.0 | 0.0 |
| Rubber | 0.0 | 0.0 | 0.0 | 51.7 | 0.0 | 0.0 | 0.0 | 0.0 | 0.0 | 0.0 |
| Other crops | 0.0 | 85.4 | 6.4 | 0.0 | 0.0 | 0.0 | 0.0 | 0.0 | 0.0 | 0.0 |
|  |  |  |  |  |  |  |  |  |  |  |

Source: Authors estimates using on information from various sources, including Africa Fertilizer (2022), Ludemann et al. (2022), and data extracted from national farm and household surveys.

Note: Not all countries grow all the crops shown in the table, or those crops are not disaggregated in the model databases (these appears as zeros in the table).
